# Critical evaluation of three cryo-EM structures of particulate methane monooxygenase by quantum refinement

**DOI:** 10.1107/S2059798325008356

**Published:** 2025-10-08

**Authors:** Gayathri Yuvaraj, Kristoffer J. M. Lundgren, Elija Veenman, Esko Oksanen, Ulf Ryde

**Affiliations:** a Department of Computational Chemistry Lund University Chemical Centre, PO Box 124 SE-221 00Lund Sweden; b European Spallation Source ESS ERIC PO Box 176 SE-221 00Lund Sweden; c LINXS Institute of Advanced Neutron and X-ray Science, Mesongatan 4, SE-224 84Lund, Sweden; Institut de Biologie Structurale, France

**Keywords:** particulate methane monooxygenase, cryogenic electron-microscopy, quantum refinement, copper

## Abstract

Particulate methane monooxygenase (pMMO) is an enzyme that converts methane into methanol at ambient temperature and pressure. Over the past three decades, the metal content and location of the active site have been highly controversial. Recent single-particle cryogenic electron-microscopy (cryo-EM) structures have furthered this debate. In this study, three cryo-EM structures (PDB entries 7s4h, 7s4j and 7ev9) are analysed by quantum refinement (QR). This approach augments traditional structural refinement with quantum-mechanical (QM) calculations for a small but interesting part of the protein (in this case, the copper sites). Our results indicate that the bis-His (Cu_A_) site is correctly modelled as a mononuclear copper site in all three structures. The His-brace (Cu_B_) site is also best modelled as mononuclear in all structures, although it was suggested to be a binuclear site in PDB entry 7ev9. The Cu_C_ site, which is observed only in PDB entry 7s4j, is correctly modelled and is probably reduced in the structure. The Cu_D_ putative active site, observed only in PDB entry 7s4h, is also mononuclear, but a water molecule might at least intermittently coordinate to the copper ion. On the other hand, our study does not find any support for the five additional copper ions suggested to be present in PDB entry 7ev9, including the suggested trinuclear active site and two sites in the so-called copper sponge. Instead, more chemically reasonable structures and better fit to both the cryo-EM and QM data are obtained if these copper ions are replaced with water molecules. This study illustrates the potential of QR as a standard component of cryo-EM studies for metal sites, for which reliable empirical restraints are missing.

## Abstract 2

We have studied the copper sites in three cryogenic electron-microscopy structures of particulate methane monooxygenase with quantum refinement. We show that the Cu_A_, Cu_C_ and Cu_D_ sites are correctly modelled and that the Cu_B_ site is mononuclear in all structures, whereas we find no support for copper in the suggested trinuclear active site or two sites in the so-called copper sponge in PDB entry 7ev9.

## 1. Introduction

Methane, the primary component of natural gas and a potent greenhouse gas (Mar *et al.*, 2022 ▸), shows promise as a transitional energy source due to its lower carbon emissions compared with traditional fossil energy sources such as oil and coal (Ritchie *et al.*, 2025 ▸; Rosselot *et al.*, 2021 ▸). However, harnessing its full potential as an energy resource presents challenges due to the high inertness of its carbon–hydrogen bonds, which are characterized by a bond dissociation energy of 435 kJ mol^−1^ (Murrell *et al.*, 2000 ▸). Efficiently converting methane into liquid methanol would improve the storage and utilization of methane. Methanol also serves as a critical building block for many industrial processes, including fuel production, polymer synthesis and chemical manufacturing. Moreover, achieving the efficient conversion of methane to methanol could help mitigate the impact of methane as a greenhouse gas.

In nature, the conversion of methane to methanol under ambient conditions is performed by methanotrophic bacteria, which employ a specialized group of enzymes known as methane monooxygenases (MMOs). MMOs can be classified into two major classes: soluble methane monooxygenases (sMMOs) and particulate methane monooxygenases (pMMOs) (Murrell *et al.*, 2000 ▸). Due to their simple purification, sMMOs have been studied extensively and are well understood, while many aspects of pMMOs are still shrouded in mystery (Hakemian & Rosenzweig, 2007 ▸).

Studying and elucidating the reaction mechanism of pMMO is anticipated to contribute greatly to the development of an efficient methane-conversion process. Therefore, a great amount of research has been dedicated to elucidating the structure, metal content and active site of pMMO (Cutsail *et al.*, 2021 ▸; Chan *et al.*, 2022 ▸). In particular, there has been a longstanding debate regarding the exact nature and location of the active site in pMMO. Several models and hypotheses have appeared over the years (Chen, Chen *et al.*, 2013 ▸; Lieberman & Rosenzweig, 2005*b* ▸; Lieberman *et al.*, 2003 ▸; Chan *et al.*, 2007 ▸; Rosenzweig, 2008 ▸).

In 1994, Chan and coworkers used electron paramagnetic resonance spectroscopy (EPR) and magnetic susceptibility to suggest that the active site of pMMO contains an exchange-coupled trinuclear copper(II) cluster (Nguyen *et al.*, 1994 ▸). Subsequently, the subunits of pMMO were isolated and the presence of 12–15 Cu ions per 94 kDa monomeric unit was reported (Nguyen *et al.*, 1998 ▸). Moreover, they used X-ray absorption-edge spectroscopy and EPR studies to distinguish catalytic and electron-transfer clusters within the enzyme (Chen, Chen *et al.*, 2013 ▸; Hung *et al.*, 2013 ▸).

**Figure 1:**
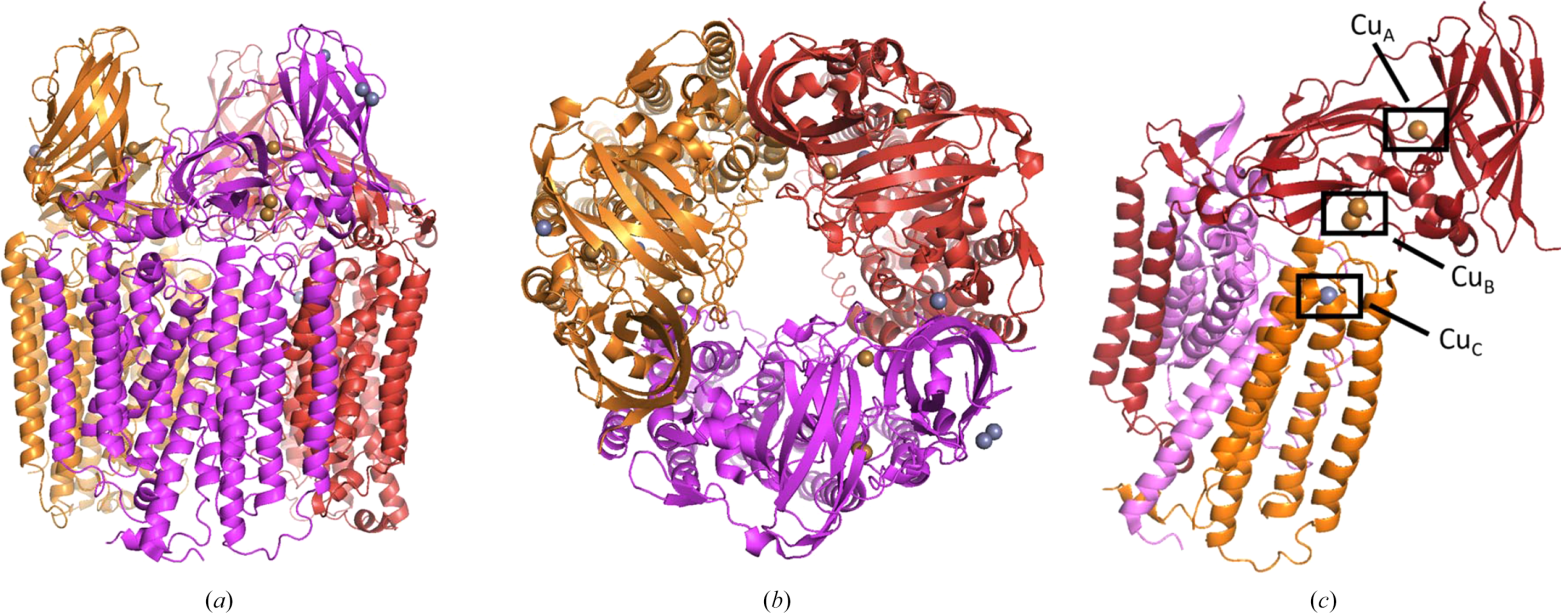
The pMMO trimer viewed from the side (*a*) and from the top (*b*) with the three protomers shown in orange, magenta and red. (*c*) One protomer of the trimeric structure with the Cu_A_ site, the Cu_B_ site and the Cu_C_ site marked. The figure is based on PDB entry 1yew (Lieberman & Rosenzweig, 2005*b* ▸).

In contrast, Rosenzweig and coworkers isolated pMMO from *Methylococcus capsulatus* and found only 4.8 ± 0.8 copper ions per 200 kDa pMMO (Lieberman *et al.*, 2003 ▸). Using extended X-ray absorption fine-structure data, they identified oxygen/nitrogen ligation and a 2.57 Å Cu–Cu interaction. In 2005, Rosenzweig and coworkers managed to crystallize pMMO and solve the structure at a resolution of 2.8 Å. It revealed a trimeric α_3_β_3_γ_3_ (PmoA, PmoB and PmoC) polypeptide arrangement (Lieberman & Rosenzweig, 2005*b* ▸). PmoA and PmoC are almost completely embedded in the membrane and consist of seven and five transmembrane helices, respectively. PmoB has a large soluble domain and consists of two cupredoxin-like β-barrels which are connected by two transmembrane helices. These three subunits constitute one protomer, and the complete complex consists of three protomers with a threefold symmetry axis (Fig. 1 ▸).

The crystal structure showed three metal sites. One copper site (Cu_A_) is located in the soluble PmoB domain and has two histidine ligands in an almost linear geometry. Gln404 is sometimes a third ligand but it is not conserved in all pMMOs from different sources (Lieberman & Rosenzweig, 2005*b* ▸). The second copper site (Cu_B_) is also within the PmoB subunit and was modelled as a dinuclear copper site with three histidine ligands. It was suggested to be the active site (Lieberman & Rosenzweig, 2005*a* ▸). The third metal centre (Cu_C_), which contained a zinc ion in the first crystal structures, is located in the PmoC subunit. It involves three highly conserved ligands: one aspartate and two histidines.

However, Chan and coworkers argued that the enzyme on which the structure was based does not display activity, indicating that it may be lacking essential copper cofactors (Yu *et al.*, 2007 ▸; Chan *et al.*, 2007 ▸). They reproduced the Rosenzweig purification procedure and observed a loss of about 12 copper ions (Chan *et al.*, 2007 ▸) per trimer. In 2008, they proposed a model of a trinuclear copper centre in the crystal structure (Cu_E_; Chan & Yu, 2008 ▸). Moreover, they provided evidence for their proposed active site and copper content in the form of theoretical modelling (Chen & Chan, 2006 ▸), redox potentiometry studies (Chan *et al.*, 2007 ▸) and model complexes (Mou *et al.*, 2018 ▸). They also built rationally designed tri­copper complexes (Chen, Nagababu *et al.*, 2013 ▸; Chan *et al.*, 2012 ▸, 2013 ▸; Nagababu *et al.*, 2012 ▸) and a tricopper-binding peptide based on the Cu_E_ site residues, which were shown to exhibit methane-oxidation activity (Chen, Nagababu *et al.*, 2013 ▸).

In the following years, Rosenzweig and coworkers solved two further crystal structures of pMMO from *Methylosinus trichosporium* (Hakemian *et al.*, 2008 ▸) and *Methylocystus* sp. (Smith *et al.*, 2011 ▸). The conservation of the dicopper site in all three structures, combined with the experimental observation that O_2_ binds to the PmoB subunit, pointed in the direction of Cu_B_ as the active site (Hakemian *et al.*, 2008 ▸; Balasubramanian *et al.*, 2010 ▸; Culpepper & Rosenzweig, 2012 ▸; Culpepper *et al.*, 2012 ▸). However, in 2018 we used quantum refinement to show that there is actually no crystallographic support for a di­nuclear Cu_B_ site; instead, much more reasonable structures were obtained with a mononuclear site (Cao *et al.*, 2018 ▸). In subsequent years, Rosenzweig and coworkers confirmed this suggestion and they also reinterpreted their previous results and suggested that the active site is instead Cu_C_ (Ross *et al.*, 2019 ▸; Ro *et al.*, 2019 ▸). New investigations showed that methane oxidation does not depend on the Cu_B_ site (Ross *et al.*, 2019 ▸). Moreover, Cu_B_ is not conserved in pMMOs from all bacterial strains (Semrau *et al.*, 2010 ▸), whereas the Cu_C_ site is conserved across all known methanotrophic species (Koo & Rosenzweig, 2020 ▸). Occupation of the Cu_C_ site by zinc instead of copper led to complete abolishment of the activity (Sirajuddin *et al.*, 2014 ▸). Moreover, top-down mass spectrometry showed that a high occupancy of Cu_C_ correlated with an increase in activity (Ro *et al.*, 2019 ▸), and nitrite, a known pMMO inhibitor, was shown to alter the Cu_C_ EPR signal in combination with decreased activity (Ross *et al.*, 2019 ▸).

In 2021, Chan and coworkers published the first single-particle cryo-EM structure of pMMO (PDB entry 7ev9) at a resolution of 2.60 Å (Chang *et al.*, 2021 ▸). They developed a method based on quantitative electrostatic potential (ESP) profiling and used it to identify eight of the expected 12–15 copper ions in the structure. This included the Cu_A_ and Cu_B_ sites, as well as two metal ions in the Cu_E_ site, whereas the Cu_C_ site was missing (Chan *et al.*, 2021 ▸).

The year after, Rosenzweig and coworkers published eight single-particle cryo-EM structures of pMMO in different types of surroundings with resolutions from 2.14 to 2.46 Å (Koo *et al.*, 2022 ▸). In all structures three copper ions per protomer were found and the activity was approximately half of that for the native membrane-bound enzyme. The Cu_A_ and Cu_B_ sites were present in all structures and showed the same ligands and geometry as in the crystal structures. In some structures a copper ion in the Cu_C_ site was observed, but in other structures the ion had moved ∼6 Å to a nearby site, which will be called Cu_D_ in the following. The latter site seems to need the stabilizing effect of the lipids to be formed (Koo *et al.*, 2022 ▸). It consists of one asparagine and two histidine ligands. The two sites were never occupied simultaneously in any of the structures. Recent quantum-mechanical studies have suggested that the Cu_D_ site is the active site of pMMO (Peng *et al.*, 2023 ▸). This has also been supported by a study of the binding of 2,2,2-trifluoroethanol (a product analogue) to pMMO by cryo-EM and ENDOR spectroscopy (Tucci *et al.*, 2023 ▸).

Given the conflicting nature of the pMMO cryo-EM structures, we herein evaluate the various copper sites in three of these cryo-EM structures. We use quantum refinement (Ryde *et al.*, 2002 ▸; Bergmann *et al.*, 2022 ▸), which has recently been extended to cryo-EM structures (Lundgren *et al.*, 2024 ▸). This enables us to take a closer look at the interpretation of the copper sites and compare the different structures based on experimental and QM quality measures.

## 2. Methods

### 2.1. Real-space quantum refinement

Structural cryo-EM refinement improves the structural model by minimizing a target function that describes how close the model is to the experimental ESP map, *T*_map_. However, macromolecular experimental data are insufficient to refine all individual parameters of the model (Afonine, Poon *et al.*, 2018 ▸). Therefore, the experimental data are supplemented by additional prior information in the form of stereochemical information (for example bond distances, angles and dihedrals), internal consistency of macromolecules and additional structural knowledge (for example similarity to known structures or secondary-structure elements) (Steiner & Tucker, 2017 ▸), *T*_restr_. In the language of computational chemistry, this is essentially a molecular-mechanics potential. Consequently, the target function is a sum of two components,
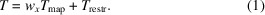
The weight factor, *w_x_*, determines the relative importance of the two terms. Several procedures to select a proper value of *w_x_* have been suggested, for example to either give the two terms a comparable magnitude or to optimize some quality criterion (Jack & Levitt, 1978 ▸; Brünger *et al.*, 1989 ▸; Brünger & Rice, 1997 ▸; van Zundert *et al.*, 2021 ▸; Afonine *et al.*, 2011 ▸).

In quantum refinement (QR; Ryde & Nilsson, 2003 ▸; Ryde *et al.*, 2002 ▸), *T*_restr_ is replaced by a quantum-mechanical (QM) calculation for a small but interesting subsystem (called region 1 or the QM region; in the present case a metal ion with its direct ligands and possibly some nearby residues). The target function then becomes
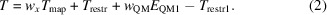


Here, *E*_QM1_ is the QM energy of the QM region and *T*_restr1_ is the restraints for the same region. Since the force field used in *Phenix* is statistical in nature, whereas the QM calculations give results in energy units, a scaling factor, *w*_QM_, is needed. Empirically, *w*_QM_ = 7.5 mol kcal^−1^ has been found to be appropriate (Lundgren *et al.*, 2024 ▸). Real-space QR is implemented in the *QRef* software (Lundgren *et al.*, 2024 ▸), which combines the *Phenix* software for real-space refinement (Liebschner *et al.*, 2019 ▸; Afonine, Poon *et al.*, 2018 ▸) and the QM interface *ORCA* (Neese *et al.*, 2020 ▸). It was thoroughly tested in our previous study (Lundgren *et al.*, 2024 ▸) and is freely available from https://github.com/krlun/QRef.

The *Phenix* refinements were performed with the *phenix.real_space_refine* module (Afonine, Poon *et al.*, 2018 ▸). We used the default parameters, except that the conformation-dependent library, secondary-structure and Ramachandran restraints were turned off. Removing such restraints is acceptable because quantum refinement is run at the end of the refinement, when the global fold and general structure are already settled, and only the detailed structure of a small part of the structure is of interest. Moreover, the surrounding structure is kept close to the starting structure by using self-reference restraints (reference_coordinate_restraints.sigma=0.01; Lundgren *et al.*, 2024 ▸). Three macrocycles of coordinate refinement were performed, followed by one macrocycle of atomic displacement parameter refinement. Figures were constructed by *PyMOL* (version 2.0, Schrödinger).

In this work, we have re-evaluated the metal sites in three single-particle cryo-EM protein structures of pMMO with PDB entries 7ev9 (Chang *et al.*, 2021 ▸), 7s4j and 7s4h (Koo *et al.*, 2022 ▸). The three structures have reported resolutions of 2.6, 2.14 and 2.16 Å, respectively. The latter two structures were selected from Rosenzweig’s eight structures because they were the structures with the best resolution that contained the Cu_C_ or Cu_D_ sites, respectively. For all three structures, coordinates, occupancies and atomic displacement parameters were obtained from the Protein Data Bank (Berman *et al.*, 2000 ▸). Electron-microscopy primary maps were downloaded from the Electron Microscopy Data Bank (EMDB): EMDB codes EMD-24826, EMD-24828 and EMD-31325, respectively (Turner *et al.*, 2024 ▸). The QM region was protonated with *phenix.ready_set* using a manual determination of the protonation state (Asp, Glu, Lys and Arg were in their charged states; His residues were protonated on the N atom not coordinating to the Cu ions). Restraint files for the non­standard ligands were generated using *phenix.elbow* (Moriarty *et al.*, 2009 ▸). Since the metal sites are modelled identically in the three protomers of the deposited structures, we restricted the QR investigations to the first metal site of each type in the structure (*i.e.* in the first protomer).

To estimate how well the QR structure fits the ESP map, we calculated the real-space correlation coefficient (RSCC) using the *Phenix* tool *phenix.map_model_cc* (Afonine, Klaholz *et al.*, 2018 ▸). The RSCC calculations involve electronic scattering factors for neutral atoms for all atoms (*Phenix* 1.21.2 does not support charged species). The RSCC is calculated for all atoms in any residue involving atoms in the QM region, based on a radius criterion. In variance to X-ray crystallography, the ESP map in cryo-EM is not model-biased, so it does not change with the model. Therefore, RSCC will always be deteriorated by any restraint term, even if it ensures that the structure is more chemically reasonable. Thus, some decrease in RSCC caused by the QM restrains is expected, as was thoroughly discussed in our previous study (Lundgren *et al.*, 2024 ▸).

All QM calculations were performed with the TPSS density-functional theory method (Tao *et al.*, 2003 ▸), D4 dispersion corrections (Caldeweyher *et al.*, 2019 ▸) and the def2-SV(P) basis set (Schäfer *et al.*, 1992 ▸). The QM region is described for each metal site below. The QM regions were truncated by hydrogen link atoms (Lundgren *et al.*, 2024 ▸). In a few calculations we observed spurious proton transfers within the QM system. These were avoided by running the QM calculations with a conductor-like polarized continuum model (CPCM) continuum solvent (Cammi *et al.*, 2000 ▸) using a dielectric constant of 4 (Bergmann *et al.*, 2021 ▸) and default values for the atomic radii and all other parameters. The time required for the quantum refinements depends on the size of the QM region and the computer used, but is typically 2–270 h on a single computer core.

To judge how close various structures are to an ideal QM structure, we calculated QM strain energies (Δ*E*_str_). These were obtained by comparing the QM energy of the QM region for the structure of interest (the deposited structure or a QR structure) with a structure in which the QM region is optimized in a vacuum (or the CPCM solvent if it was used for QR). To avoid the movement of protein residues to form a structure that is qualitatively different from that found in the protein, the hydrogen link atoms were kept fixed during the optimization (Lundgren *et al.*, 2024 ▸). For the deposited structure, H atoms were added with *phenix.ready_set* (as for the QR structures) and then optimized by QM, keeping the coordinates of the heavy atoms fixed at the coordinates of the deposited structure. Δ*E*_str_ depends on the oxidation state of the copper ion, so several strain energies are reported for the deposited structure with different oxidation states. It should be noted that the strain energy increases with the size of the QM region and the absolute charge of the model. Therefore, it is not comparable between the different copper sites.

When comparing various interpretations of a certain metal site, it is necessary to use the same value of the *w*_*x*_ weight factor in equation (1) for all structures. As in our previous investigation (Lundgren *et al.*, 2024 ▸), we determined the ideal value of *w*_*x*_ by obtaining QR structures for ten different values of *w*_*x*_ between 0 and 100. The ideal value was selected as the highest value before the strain energy started to increase and the average RSCC of residues in the QM region started to decrease. The results of these QR calculations are shown in Supplementary Table S1. The resolution of cryo-EM structures can differ in different parts of the structure and the ideal weight can thus also differ. Therefore, different values were employed for the various metal sites in the three cryo-EM structures. In practice, the difference was small (*w*_x_ was either 3 or 10 for all structures and metal sites). In general, this selected value is close to the average value of *w*_*x*_ suggested by *Phenix* (different values are suggested for each macrocycle; the ideal value suggested by *Phenix* varied between 1.0 and 6.6) and in future applications the value suggested by *Phenix* can safely be used (but it is important to use the same fixed value in all calculations of different interpretations of the structure).

### 2.2. CheckMyMetal

To obtain an independent opinion on whether the various metal-binding sites in the cryo-EM structures are chemically reasonable, we used the *CheckMyMetal* (*CMM*) webserver (Zheng *et al.*, 2014 ▸). *CMM* diagnoses metal-binding sites in protein structures based on seven quality measures and each measure is judged to be either acceptable, borderline or dubious. The thresholds are based on a statistical evaluation of metal sites in protein crystal structures (Zheng *et al.*, 2017 ▸).

## 3. Results and discussion

We have studied the metal sites in three recent cryo-EM structures of pMMO (PDB entries 7s4j, 7s4h and 7ev9) using QR. We will discuss the various metal sites in separate sections.

### 3.1. The Cu_A_ site

The Cu_A_ site (often called the bis-His site) is the least controversial metal site in pMMO. It is present in all three cryo-EM structures and is modelled in a similar way (Chang *et al.*, 2021 ▸; Koo *et al.*, 2022 ▸). They only differ in whether the OE1 atom of Gln404A is within bonding distance or not. Since this site is uncontroversial, we start with this site to set a precedent for later comparisons.

**Figure 2:**
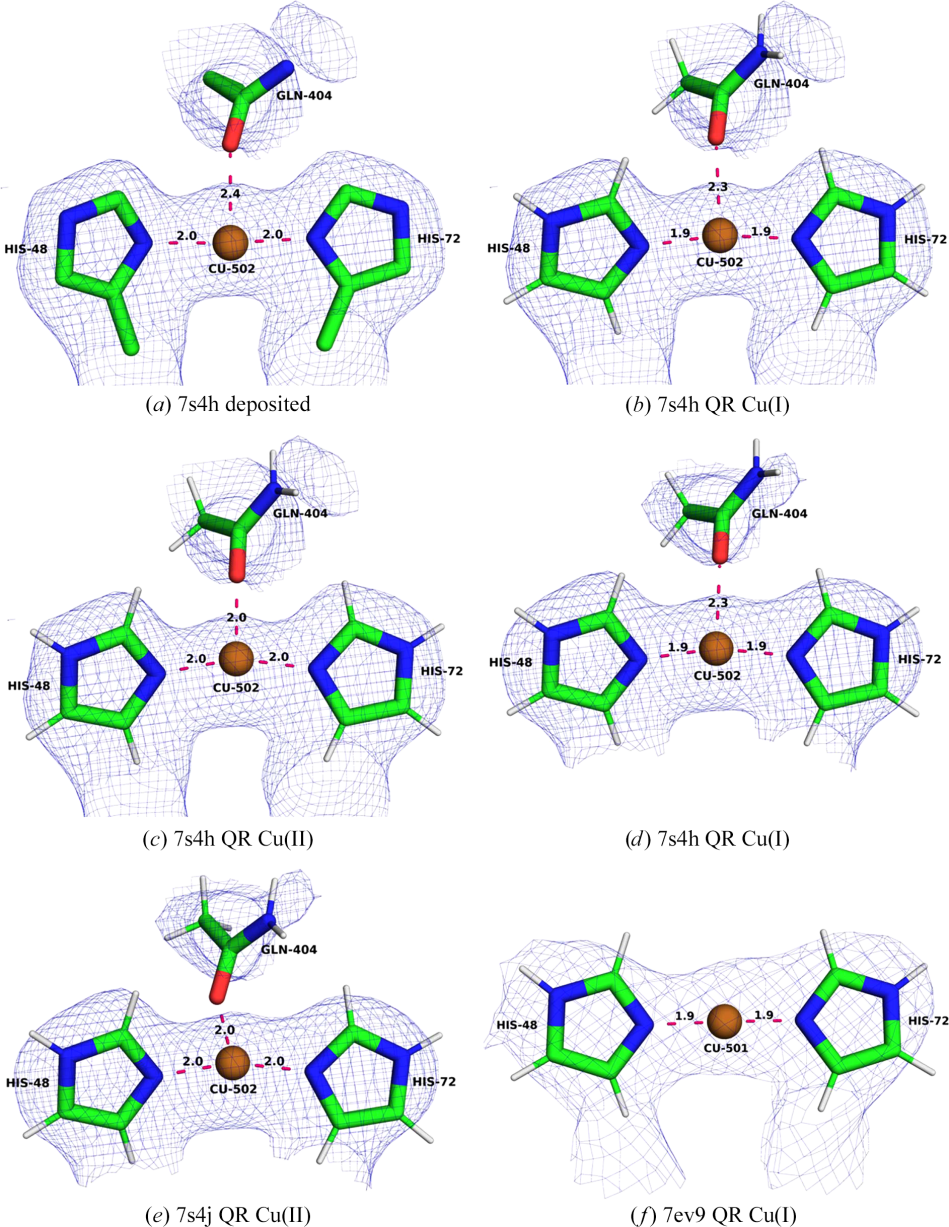
The bis-His (Cu_A_) site in the various structures. (*a*) The deposited PDB entry 7s4h structure, (*b*) 7s4h QR with Cu(I), (*c*) 7s4h QR with Cu(II), (*d*) 7s4h QR with Cu(I), (*e*) 7s4j QR with Cu(II) and (*f*) 7ev9 QR with Cu(I). The cryo-EM maps are shown at a 0.3 threshold.

In the deposited PDB entry 7s4h (Fig. 2 ▸*a*), the Cu_A_ site is mononuclear and tricoordinate with two ND1 atoms from His48A and His72A (the letter after the residue number specifies the chain ID in the PDB file), as well as the OE1 atom of Gln404A (in the following, we will call the coordinating atoms N1, N2 and O, where 1 is the lower residue number and 2 is the higher). The Cu–N1, Cu–N2 and Cu–O distances are 2.03, 2.04 and 2.41 Å, respectively. We performed two QR calculations, one with a reduced Cu(I) ion and one with an oxidized Cu(II) ion (Figs. 2 ▸*b* and 2 ▸*c*). In both QR structures, the copper–ligand distances shorten. For Cu(I) we obtain Cu–N1 and Cu–N2 distances of 1.91 and 1.92 Å, respectively, and the Cu–O distance is 2.34 Å. For Cu(II) we obtain Cu–N1 and Cu–N2 distances of 1.95 and 1.97 Å, respectively, and a Cu–O bond length of 2.02 Å. Even though this site is modelled well and only a small movement of the copper ion is observed, the QR structures reproduce the experimental data slightly worse than the deposited structure (Table 1 ▸). The average RSCC (over the four residues in the QM region) decreases from 0.90 for the deposited structure to 0.88 for Cu(I) and 0.87 for Cu(II). On the other hand, the strain energies decrease from 101 to 15 kJ mol^−1^ for Cu(I) and from 148 to 37 kJ mol^−1^ for Cu(II). Both RSCC and Δ*E*_str_ indicate that the Cu_A_ site is in the reduced state in the cryo-EM structure, as is also supported by the low coordination number and the long bond to Gln404.

In the deposited PDB entry 7s4j, the Cu_A_ site is also mononuclear and tricoordinate. The two Cu–N distances are 1.95 and 1.97 Å, respectively, and the Cu–O distance is 2.38 Å, *i.e.* all distances are slightly shorter than in PDB entry 7s4h. Again, we performed QR for both the Cu(I) and Cu(II) states (Figs. 2 ▸*d* and 2 ▸*e*). The results are also included in Table 1 ▸. For both oxidation states the Cu–N distances decrease slightly. For Cu(I), the Cu–N distances are 1.91–1.93 Å, which are almost identical to those in PDB entry 7s4h. For Cu(II) both Cu–N distances are 1.96 Å. The Cu–O bond length decreases even more, to 2.31 Å for Cu(I) and to 1.98 Å for Cu(II), which are slightly shorter than for PDB entry 7s4h. The average RSCC of the deposited structure is 0.90, whereas it is 0.88 for Cu(I) and 0.86 for Cu(II) of the QR structures. The strain energy decreases from 84 kJ mol^−1^ for the deposited structure to 14 kJ mol^−1^ in the QR structure for Cu(I) and from 134 to 42 kJ mol^−1^ for Cu(II). Again, the results indicate that the Cu_A_ site is reduced in the cryo-EM structure.

In PDB entry 7ev9, the Cu_A_ site is also mononuclear, but is two-coordinate since the Gln404A OE1 atom is found at a distance of 5.2 Å. The Cu–N1 and Cu–N2 bonds are 1.90 and 1.87 Å, respectively. We performed QR for Cu(I) and Cu(II) ions and found slightly compressed bond lengths. For Cu(I) (Fig. 2 ▸*f*), Cu–N1 and Cu–N2 are 1.89 and 1.90 Å, respectively. For Cu(II), both Cu–N1 and Cu–N2 are 1.91 Å. Thus, both Cu–N distances are slightly shorter than in the other two cryo-EM structures, reflecting the decrease in coordination number. Like the other two structures, the RSCC values for the QR structures are slightly lower than for the deposited structure, 0.91, compared with 0.92. The strain energies for the QR structures are 4–5 kJ mol^−1^, lower than for the deposited structure, 36–38 kJ mol^−1^. The strain energies are appreciably lower than for the other two cryo-EM structures, reflecting that the QM system now includes only two ligands (*i.e.* there are fewer atoms that can be strained).

These results confirm that the site is correctly modelled in all three cryo-EM structures. QR improves the structure slightly but does not change anything drastically. The difference between the deposited model and the QR models is small, but the RSCC scores decrease slightly for the QR structures. These results show the results that can be expected from QR when the site is correctly modelled.

### 3.2. The Cu_B_ site

Next, we study the Cu_B_ site (often called the histidine-brace site). As discussed in Section 1, this site was originally modelled as a dicopper site (Lieberman *et al.*, 2003 ▸; Lieberman & Rosenzweig, 2005*b* ▸), but it was later reinterpreted as mononuclear (Cao *et al.*, 2018 ▸; Ross *et al.*, 2019 ▸; Ro *et al.*, 2019 ▸). Interestingly, the Cu_B_ site is mononuclear in Rosenzweig’s cryo-EM structures (PDB entries 7s4j and 7s4h; Koo *et al.*, 2022 ▸), whereas it is dinuclear in PDB entry 7ev9 (Chang *et al.*, 2021 ▸).

**Figure 3:**
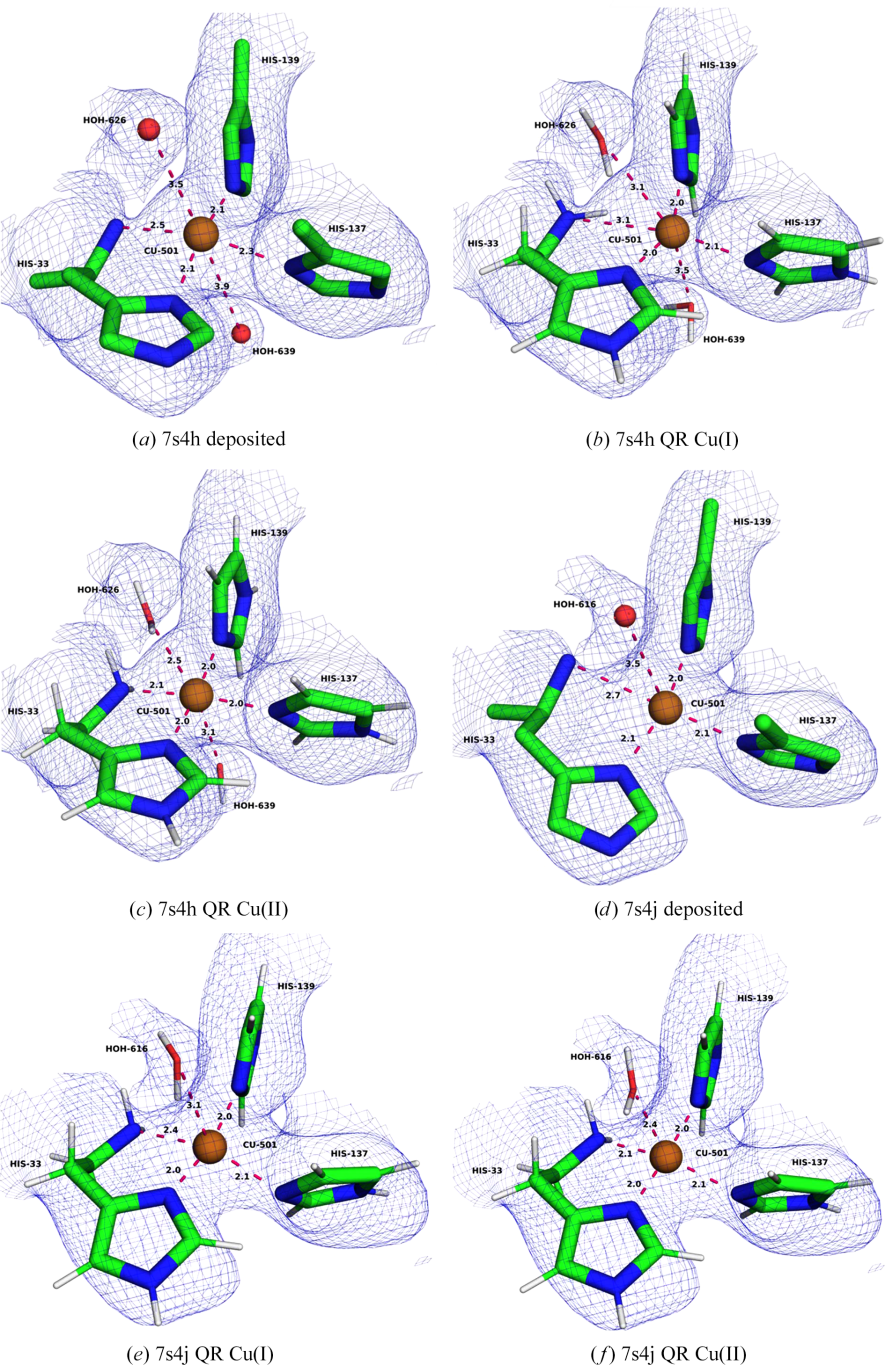
The Cu_B_ site in PDB entries 7s4h and 7s4j. (*a*) The deposited PDB entry 7s4h structure, (*b*) 7s4h QR with Cu(I), (*c*) 7s4h QR with Cu(II), (*d*) the deposited PDB entry 7s4j structure, (*e*) 7s4j QR with Cu(I) and (*f*) 7s4j QR with Cu(II). The maps are shown at a 0.3 threshold.

In PDB entry 7s4h, the Cu_B_ site is mononuclear and four-coordinate with three N atoms from the side chains of His33A, His137A and His139A, as well as the amino-terminal N atom of His33A (Fig. 3 ▸*a*; these N atoms will be called N2, N3 and N4 as well as N1 in the following, reflecting their order in the PDB file). The Cu–N distances are 2.06–2.29 Å for N2–N4 and 2.55 Å for Cu–N1. There are also two water molecules with Cu–O distances of 3.5–3.9 Å.

We ran QR calculations with either Cu(I) and Cu(II) ions (Figs. 3 ▸*b* and 3 ▸*c*). In the oxidized structure, all four N atoms coordinate to Cu with Cu–N distances of 2.00–2.02 Å for the side-chain atoms and 2.08 Å for N1. Moreover, one of the water molecules coordinates to the copper ion with a Cu–O distance of 2.54 Å and the distance to the other water molecule also decreases to 3.12 Å. This reflects that the two water molecules do not have anything else to interact with. It also reflects that Cu(II) typically forms square-planar geometries, possibly with one or two weaker axial ligands. This structure is supported by the RSCC score of one of the water molecules, which increases from 0.69 to 0.74, whereas the other remains at 0.58 (Supplementary Table S3). On the other hand, the RSCC of the copper ion decreases slightly from 0.91 to 0.87. The average RSCC for the six residues in the QM region decreases from 0.81 to 0.80 in the QR structure (Table 2 ▸). The strain energy, 26 kJ mol^−1^, is much lower than in the deposited structure (204 kJ mol^−1^).

The QR structure of the reduced Cu(I) state is quite different. Only N2–N4 coordinate to copper with Cu–N distances of 1.97–2.06 Å, whereas the Cu–N1 and the two Cu–O distances are long at 3.08–3.48 Å (but the Cu–O distances are still shorter than in the deposited structure). The average RSCC is slightly worse than in the oxidized structure (0.79), in particular for the two water molecules and the copper ion. The strain energy is also appreciably higher at 93 kJ mol^−1^, although it is smaller than in the deposited structure (183 kJ mol^−1^). Consequently, the QR results indicate that the Cu_B_ site is oxidized in the cryo-EM structure.

In the deposited PDB entry 7s4j, the Cu_B_ site is also mononuclear and four-coordinate (Fig. 3 ▸*d*). There is a single water molecule at a nonbinding Cu–O distance of 3.46 Å. The Cu–N distances to the imidazole groups are 2.04–2.11 Å, *i.e.* rather similar to those in PDB entry 7s4h. The Cu–N1 distance to the amino-terminus is longer at 2.75 Å. Again, we performed QR with both Cu(I) and Cu(II). In the oxidized structure (Fig. 3 ▸*f*), all four N atoms coordinate to copper with Cu–N distances of 1.97–2.14 Å and the water molecule moves to a rather short distance of 2.39 Å. In the reduced QR structure (Fig. 3 ▸*e*), the three imidazole atoms still coordinate to copper at similar distances, 1.97–2.13 Å, but the distance to the N-terminus is longer at 2.36 Å. The water molecule is at a nonbonding distance (Cu–O = 3.05 Å). Both QR structures reproduce the cryo-EM data slightly worse than the deposited structure. The average RSCC of the five residues in the QM system is 0.86 for the deposited structure but 0.81 for the two QR structures (Table 2 ▸). The RSCC for copper is better in the reduced structure, but that of water is better in the oxidized structure. As usual, the strain energies of the QR structures, 44–85 kJ mol^−1^, are appreciably smaller than those for the deposited structure (156–159 kJ mol^−1^). The larger strain energy of the reduced structure is caused by the nonbonding water molecule, which moves to a position where it forms more favourable hydrogen bonds in the QM-optimized structure.

**Figure 4:**
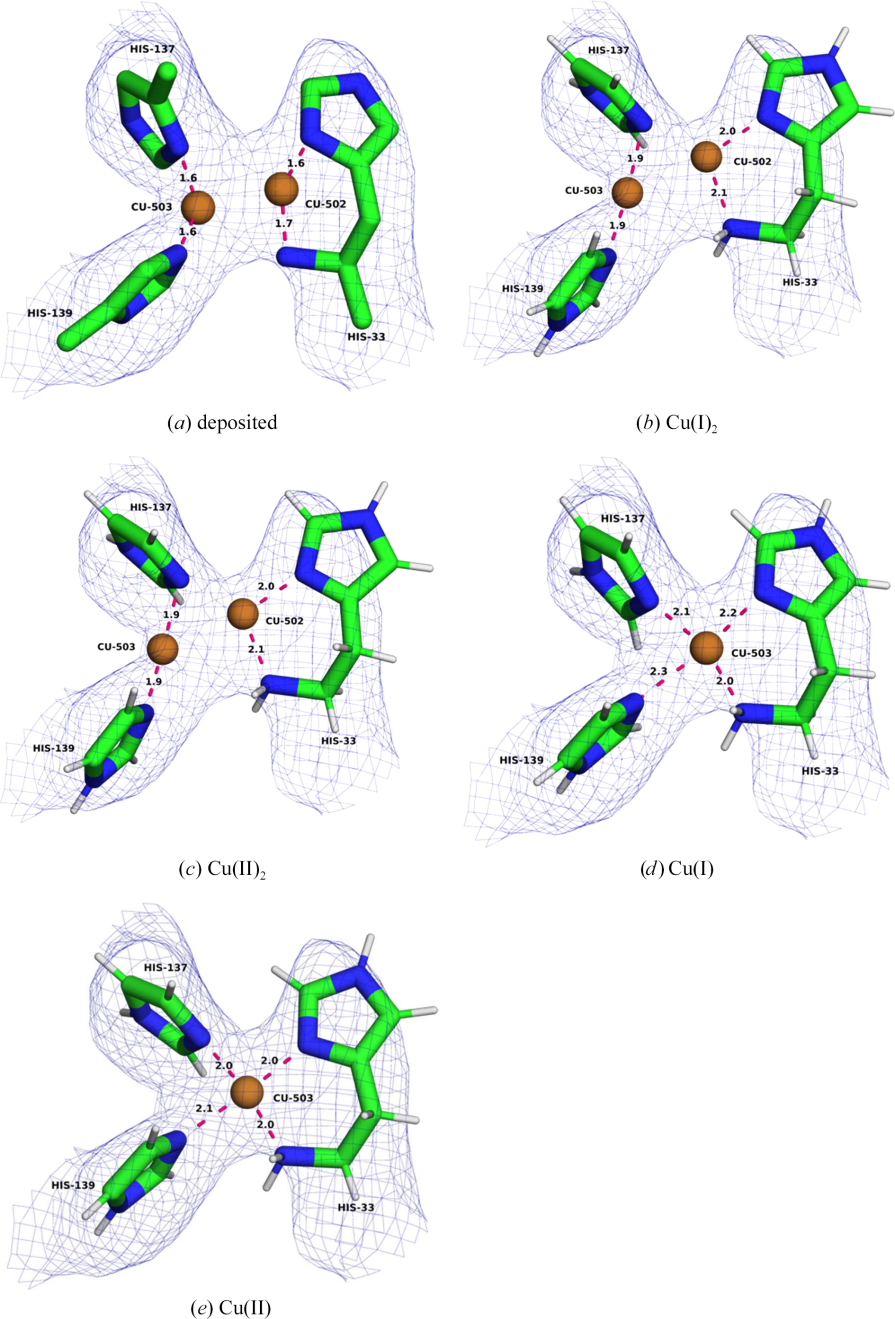
Various structures of the Cu_B_ site in PDB entry 7ev9. (*a*) The deposited structure, (*b*) QR with both coppers in the Cu(I) state, (*c*) QR with both coppers in the Cu(II) state, (*d*) QR with one copper in the Cu(I) state and (*e*) QR with one copper in the Cu(II) state. The maps are shown at a 0.3 threshold.

In contrast, Cu_B_ is modelled as a binuclear site in PDB entry 7ev9, showing a strange structure with a Cu–Cu distance of 2.27 Å and four very short Cu–N distances of 1.59–1.66 Å (Fig. 4 ▸*a*). *CMM* indicates that both copper ions have questionable geometry, with seven borderline and three dubious measures (see Supplementary Table S11). QR with two copper ions [we tested three different cases, Cu(I)_2_, Cu(I)Cu(II) and Cu(II)_2_] make the Cu–N distances more realistic (1.85–2.07 Å). The Cu–Cu distance elongates slightly to 2.38–2.41 Å and the Cu502–N3 distance decreases from 3.01 to 2.10–2.14 Å, making this copper ion tricoordinate (Figs. 4 ▸*b* and 4 ▸*c*). In addition, the copper ions move more into the planes of the histidine rings, although the structure is still far from chemically ideal. As expected, the RSCC decreases from 0.85 in the deposited structure to 0.77 in the QR structures, because Cu502 moves towards the edge of the density and His33A moves out of density (Table 2 ▸). The strain energies are 43–67 kJ mol^−1^, indicating that the structures are not optimal. Moreover, the strain energies are underestimated because the link atoms are fixed, not allowing larger changes in the structure. There are also too few ligands to fulfil the coordination preferences of Cu(II).

Therefore, we instead modelled the structure as a mononuclear site (either reduced or oxidized). We then obtain in-plane binding of the copper ion and much more reasonable binding distances and angles (Figs. 4 ▸*d* and 4 ▸*e*). With Cu(II), the Cu–N distances are 1.97–2.14 Å, the RSCC is 0.77 and the strain energy is 27 kJ mol^−1^, which is appreciably lower than for the dinuclear structures. For Cu(I), the Cu–N distances are more dissimilar, 1.97–2.33 Å, reflecting the weaker bonding and a preference for a lower coordination number of the reduced metal. The RSCC is slightly better at 0.79, but the strain energy is high at 89 kJ mol^−1^. Undoubtedly, the Cu_B_ site is also mononuclear in PDB entry 7ev9. The lower strain energy indicates that it is best modelled with Cu(II).

### 3.3. The Cu_C_ and Cu_D_ sites

As discussed in Section 1, some of Rosenzweig’s cryo-EM structures contain the Cu_C_ site, whereas in some of the other structures the Cu_D_ site is instead observed (Koo *et al.*, 2022 ▸). In none of the structures are Cu_C_ and Cu_D_ occupied simultaneously. Among the three cryo-EM structures that we have considered, Cu_C_ is observed in PDB entry 7s4j and Cu_D_ is observed in PDB entry 7s4h, whereas neither copper ions nor any of the coordinating residues of the Cu_C_ and Cu_D_ sites are observed in PDB entry 7ev9.

**Figure 5:**
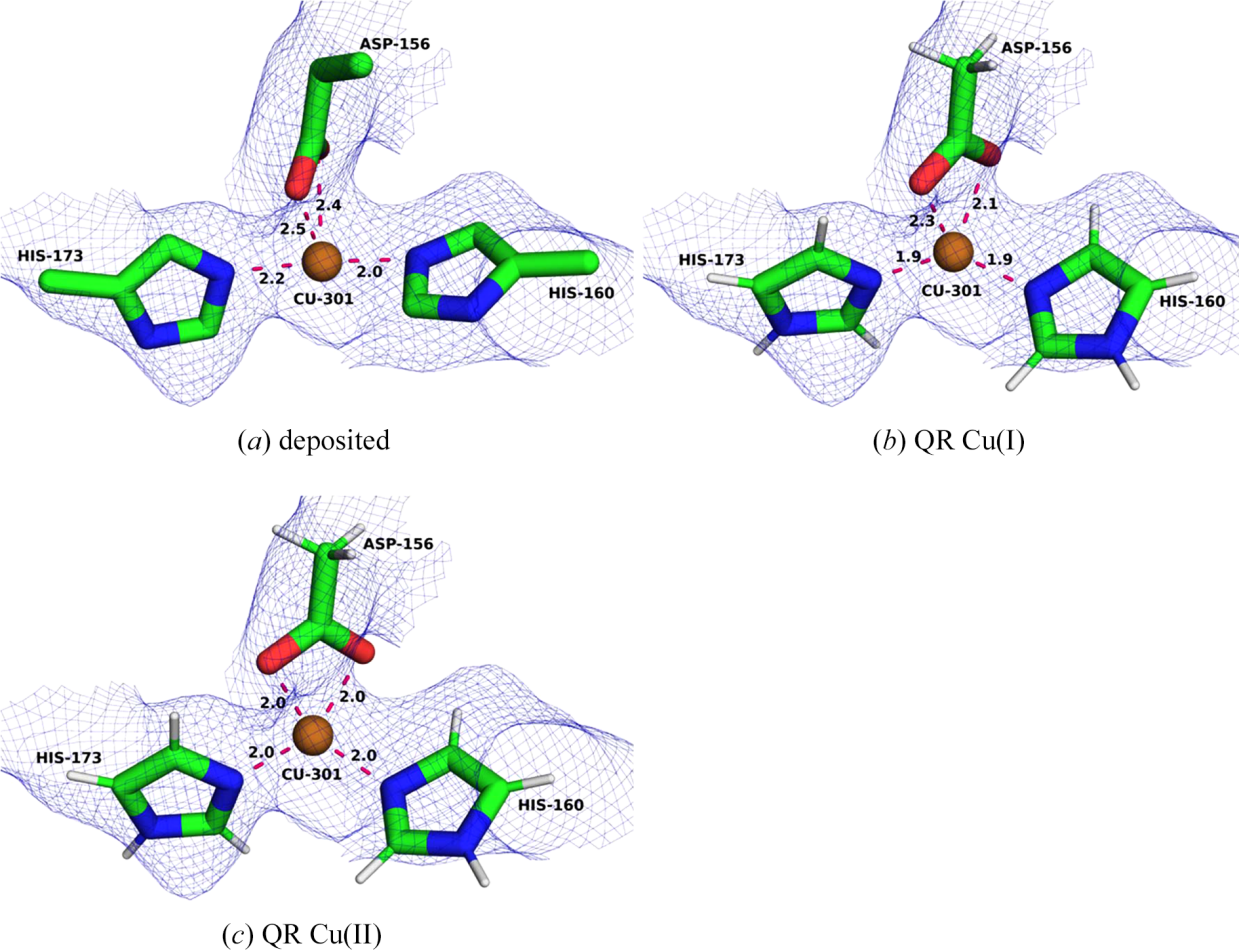
The Cu_C_ site in PDB entry 7s4j: (*a*) the deposited structure, (*b*) QR with Cu(I) and (*c*) QR with Cu(II). The maps are shown at a 0.3 threshold.

We started by studying Cu_C_ in PDB entry 7s4j. In the deposited structure, Cu301 is bound to His160C (1.99 Å), His173C (2.19 Å) and both OD atoms of Asp156C (2.51 and 2.36 Å; Fig. 5 ▸*a*). We performed QR of this site with either Cu(I) or Cu(II). The results are shown in Table 3 ▸. For both structures the Cu–O distances decreased, more for Cu(II) (2.00–2.05 Å) than for Cu(I) (2.15–2.32 Å) (Figs. 5 ▸*b* and 5 ▸*c*). Consequently, the Cu–N distances are shorter for Cu(I) (1.93 and 1.95 Å) than for Cu(II) (1.97–1.99 Å). As usual, the average RSCC values decreased slightly for the QR structures: from 0.83 for the deposited structure to 0.80 for Cu(I) and 0.78 for Cu(II). This is reflected in the ESP maps in Fig. 5 ▸, where some of the groups move somewhat out of the map, although the impression depends on the contour level of the map and the interpretation is not trivial. The decrease in RSCC and the difference between the reduced and oxidized structures are largest for copper, whereas the RSCC is actually improved for Asp156C in both QR structures, although the carboxyl group seemingly rotates out of the map in the QR structures. The strain energy is also smaller for the reduced structure (7 kJ mol^−1^) than for the oxidized structure (13 kJ mol^−1^), indicating that the Cu_C_ site is reduced in the cryo-EM structure.

**Figure 6:**
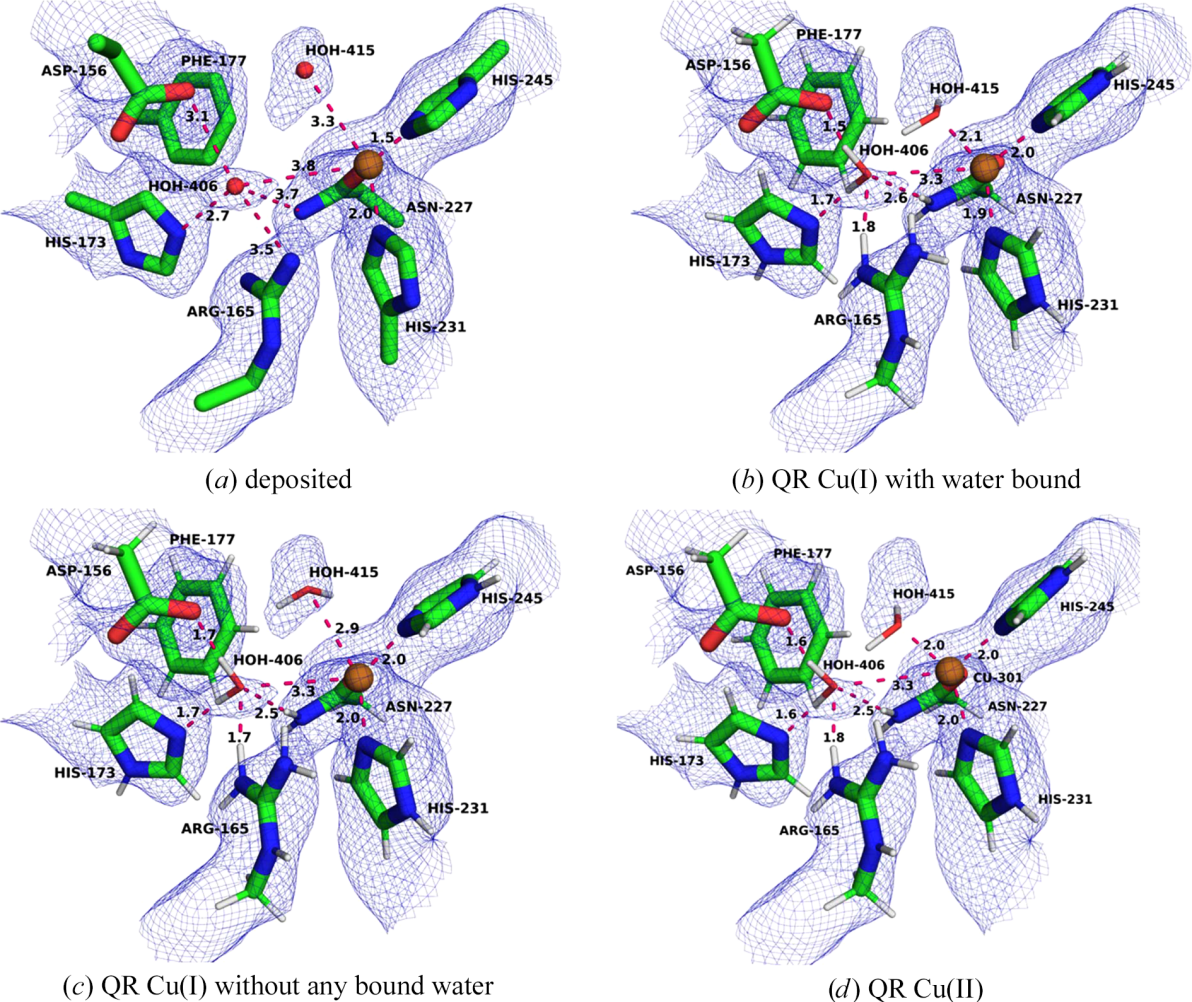
The Cu_D_ site in PDB entry 7s4h: (*a*) the deposited structure, (*b*) QR with Cu(I) and water bound, (*c*) QR with Cu(I) and without water bound and (*d*) QR with Cu(II). The maps are shown at a 0.3 threshold.

Next, we studied the Cu_D_ site in PDB entry 7s4h. In the deposited model, the copper ion is bound to OD1 of Asn227C (2.21 Å), NE2 of His231C (2.00 Å) and NE2 of His245C (1.50 Å). There are also two nearby water molecules at non­bonding distances of 3.81 and 3.30 Å (Fig. 6 ▸*a*). Clearly, the Cu–N distance to His245 is unrealistically short. This site [in the Cu(I) state] was thoroughly investigated in our previous study with three different sizes of the QM region and with and without a continuum solvent model in the QM calculations (to improve convergence and avoid spurious proton transfer; Lundgren *et al.*, 2024 ▸). Here, we only discuss the results for the largest QM region with the CPCM implicit solvent model (with a dielectric constant of 4), comparing the results for Cu(I) and Cu(II). In addition to the copper ion and its three ligands, the QM region also includes Asp156C, Arg165C, His173C and Phe177C. The former three residues can form hydrogen bonds to one of the two water molecules, whereas Phe177C provides sterical restrictions.

As expected, all QM calculations correct the Cu–N distance of His24C to 1.96–2.05 Å (Table 4 ▸). For Cu(II), the two Cu–N distances are 1.96–2.01 Å and the Cu–O distance is 2.09 Å. However, one of the water molecules also moves to coordinate the copper ion with a distance of 1.98 Å. The reason for this is that it does not form any hydrogen bonds to any protein residue and the experimental density is quite weak. QR will then move it to the closest chemically reasonable position, which is in this case coordinating to copper, also reflecting that Cu(II) prefers more than three ligands. However, the water molecule then moves out of the ESP map (Fig. 6 ▸*d*). The other water molecule forms three strong hydrogen bonds to the surrounding residues, 1.58 Å to Asp156C, 1.84 Å to Arg165C and 1.65 Å to His173C, and also a hydrogen bond to the other water molecule (1.53 Å). The strain energy is rather high, 82 kJ mol^−1^, but this mainly reflects that the QM region is large and involves three charged moieties.

For Cu(I) we could obtain two different structures, one with the water molecule coordinating to copper (2.06 Å) and the other with a Cu–O distance of 2.91 Å. The former has the lower strain energy (91 kJ mol^−1^ compared with 149 kJ mol^−1^), but also the lower average RSCC score (0.82 and 0.83). This is reflected in Figs. 6 ▸(*b*) and 6 ▸(*c*), showing that the water molecule is more outside the map for the former structure (and the RSCC for the water molecule is also appreciably lower: 0.64 compared with 0.74). However, at a lower threshold, there is also an ESP peak approaching the former water position. This may indicate that the water molecule alternates between the two positions in the cryo-EM experiment.

### 3.4. Chan active site (Cu_E_)

**Figure 7:**
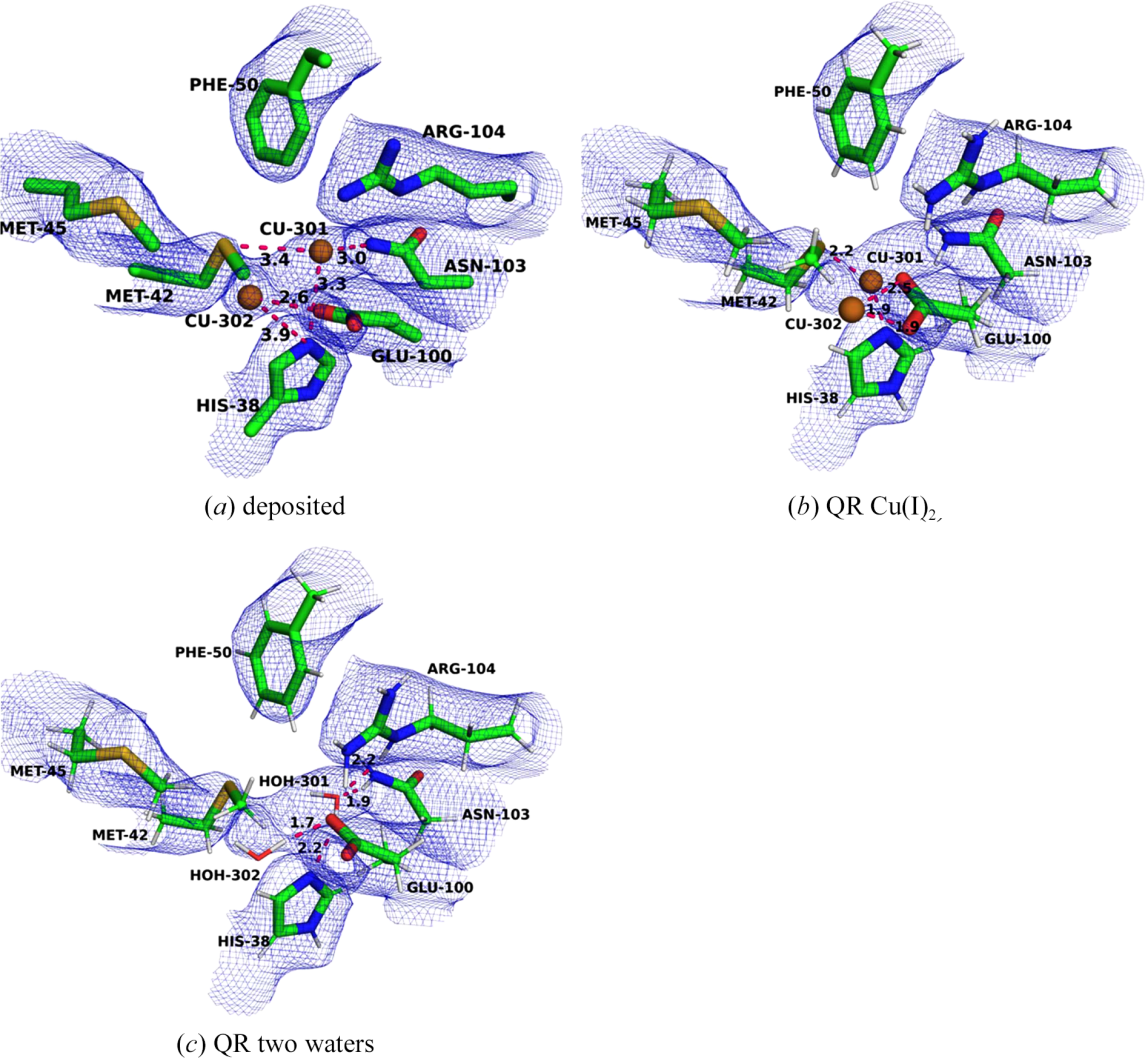
The Cu_E_ site in PDB entry 7ev9: (*a*) the deposited structure, (*b*) QR with Cu(I)_2_ and (*c*) QR with two waters. The maps are shown at a 0.3 threshold.

Since 1994, Chan and coworkers have argued that that the pMMO active site is a trinuclear copper centre in the PmoA subunit (Nguyen *et al.*, 1994 ▸). Until the publication of the cryo-EM structure (PDB entry 7ev9), it had not been observed in any experimental structure. In the deposited structure, the Cu_E_ site harbours two copper ions with a Cu–Cu distance of 4.22 Å. None of the copper ions have any ligands at normal coordinating distances. However, there are several potential coordinating atoms at larger distances: NE2 of His38B (3.28 Å to Cu301 and 3.86 Å to Cu302), SD of Met42B (3.37 and 3.64 Å), OD1 of Asn103B (2.95 Å to Cu301) and OE2 of Glu100B (2.58 Å to Cu302). On the other hand, CE of Met42B is only 1.89 Å from Cu302 and NH1 of Arg104B is 3.75 Å from Cu301. Compared with the Cu_A_ and Cu_B_ sites, the copper densities are weaker and lack convincing bonding densities (Fig. 7 ▸*a*). The *CMM* server indicates that the copper sites are questionable, with seven dubious and five borderline quality criteria.

We first performed QR with two copper ions, testing three different oxidation states [Cu(I)_2_, Cu(I)Cu(II) and Cu(II)_2_]. The results are shown in Table 5 ▸. In all three structures, Cu301 moves into binding distance of His38 (1.90–1.95 Å) and Met42 (2.21–2.29 Å) (Fig. 7 ▸*b*). Likewise, Cu302 moves towards Glu100. For the mixed-valence [Cu(I)Cu(II)] state, Glu100 binds bidentately, with Cu–O distances of 1.99 and 2.13. For Cu(I)_2_ the two distances are more dissimilar, 1.93 and 2.51 Å. For Cu(II)_2_, both a monodentate (1.89 and 3.32 Å) and a bidentate structure (1.90 and 2.30 Å) are obtained. The Cu–Cu distance increases to 4.29 (monodentate structure) or 5.38–5.59 Å (bidentate structure). However, the copper ions move out of density, as is illustrated by strongly decreasing RSCC scores (from 0.70 to 0.37–0.42 for Cu301 and from 0.67 to 0.29–0.38 for Cu302, but 0.58 for the monodentate structure). The strain energies are very high at 397–689 kJ mol^−1^ (highest for the monodentate structure), indicating that the structure is not reasonable.

Therefore, we tried to replace Cu302 with a water molecule. This did not change the structure much. Cu301 still coordinates to His38B and Met42B (1.89–1.93 and 2.23–2.24 Å), but with a far-from-ideal structure (owing to the CE methyl group of Met42B (Table 5 ▸). The water molecule forms a single hydrogen bond to Glu100B (H–O distances of 1.72–1.92 Å; both O–O distances of 2.69 Å). The average RSCC is 0.70 and the strain energies are 355–486 kJ mol^−1^, indicating a quite poor model.

If we instead replace Cu301 with a water molecule, the structure improves slightly. The copper ion forms a strong bond to OE1 of Glu100B (1.82–1.83 Å). However, it is close to the CE group of Met42B (2.13–2.20 Å, giving H–Cu distances of 2.00–2.33 Å). The water molecule forms four hydrogen bonds to NE2 of His38B (2.07–2.08 Å), ND2 of Asn103B (2.30–2.45 Å) and Arg104B (two hydrogen bonds of 2.28–2.48 and 2.41–2.60 Å); the corresponding heavy-atom distances are 3.05–3.07, 3.26–3.41, 3.16–3.33 and 3.45–3.46 Å. The distance to the copper ion is 3.66–3.71 Å. The average RSCC score is better than for the other structures at 0.76, but the strain energy is still very high at 404–754 kJ mol^−1^.

Finally, we tried to replace both copper ions with water molecules. This gave the chemically most reasonable structure (Fig. 7 ▸*c*). The water replacing Cu301 still forms hydrogen bonds to NE2 of His38B (2.16 Å), ND2 of Asn103B (1.89 Å) and Arg104B (only one of 2.16 Å, because the NH1 atom forms a hydrogen bond to Glu100B). The other water molecule forms only a single hydrogen bond to OE1 of Glu100B (1.73 Å). The corresponding heavy-atom distances are 2.97, 2.89, 3.15 and 2.72 Å. The distance between the two water molecules is 4.93 Å (O–O distance). The average RSCC score is slightly lower than for the structures with only Cu301 replaced with water (0.73), but the strain energy is much lower (208 kJ mol^−1^) and the structure is much more reasonable. Therefore, this is our best interpretation of the structure, although it is not completely satisfying, since one water molecule forms only a single hydrogen bond.

### 3.5. Cu504

**Figure 8:**
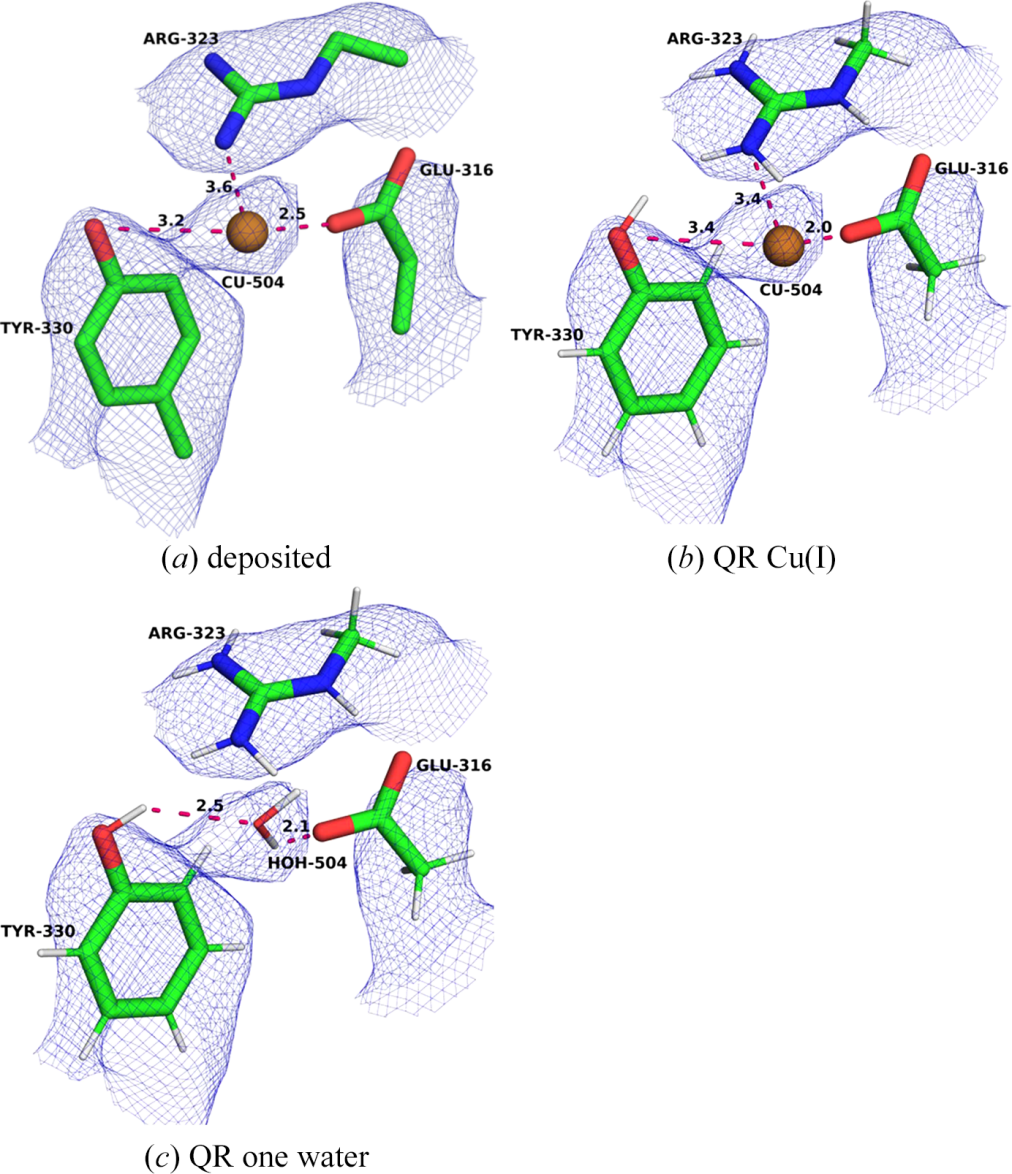
The Cu504 site in PDB entry 7ev9: (*a*) the deposited structure, (*b*) QR with Cu(I) and (*c*) QR with the copper ion replaced by a water molecule. The maps are shown at a 0.3 threshold.

PDB entry 7ev9 contains two additional copper sites that are not present in the any of Rosenzweig’s cryo-EM structures. One of them is Cu504, which is part of the so-called copper sponge (Yu *et al.*, 2007 ▸; Lu *et al.*, 2019 ▸). In the deposited structure the copper ion has only a single ligand: OE1 of Glu316A (2.51 Å; Fig. 8 ▸*a*). The other OE2 atom is turned away from the copper ion with a distance of 4.41 Å. OH of Tyr330A is 3.22 Å away, but in a noncoordinating geometry. The NH2 atom of Arg323A is only 3.57 Å away, which is unexpected for a positively charged residue. Thus, the structure does not look like a regular copper site. This is also confirmed by *CMM*, which suggest that it is most likely incorrectly modelled (four quality criteria suggest that the site is dubious and one is borderline; two measures are not available for a site with a single ligand; Supplementary Table S11). The copper density is weak compared with those of the Cu_A_, Cu_B_, Cu_C_ and Cu_D_ sites.

We first performed two QR calculations with Cu(I) or Cu(II) (Table 6 ▸). Naturally, this could not improve the structure significantly. The copper ion moves somewhat to obtain an improved Cu–O distance: 1.97 Å for Cu(I) and 2.09 Å for Cu(II). However, this moves the copper slightly out of the density (Fig. 8 ▸*b*). Consequently, the RSCC is reduced somewhat (0.72–0.73) compared with the deposited model (0.79). The strain energies are very high (248–305 kJ mol^−1^) and only slightly smaller than those of the deposited structure (384–398 kJ mol^−1^). This indicates that this site is incorrectly modelled.

Therefore, we instead tried to replace the copper ion by a water molecule. This gave a chemically much more reasonable structure. The water molecule stays inside the density and forms two hydrogen bonds (Fig. 8 ▸*c*) to Glu316 at 2.06 Å and to Tyr330 at 2.49 Å (with an enlarged QM system, a hydrogen bond to Arg40 can also be obtained). This indicates that this site is better modelled by a water molecule than by a copper ion. In fact, the RSCC is the highest among the three QR jobs (0.75). The strain energy is also smaller than for the other models at 108 kJ mol^−1^.

### 3.6. Cu505 and Cu506

**Figure 9:**
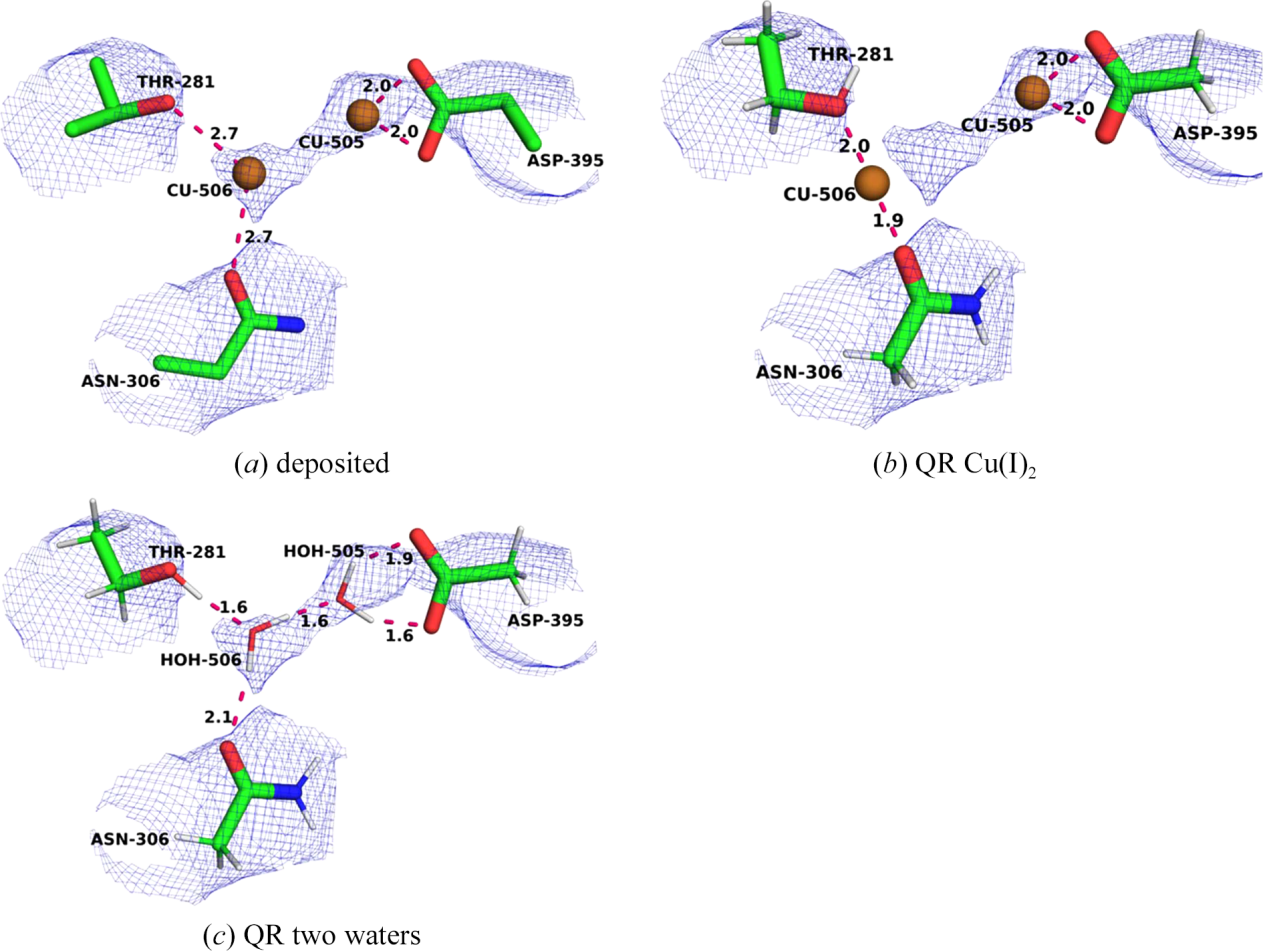
The Cu505/Cu506 site in PDB entry 7ev9: (*a*) the deposited structure, (*b*) QR with Cu(I)_2_ and (*c*) QR with both copper ions replaced with waters. The maps are shown at a 0.3 threshold.

PDB entry 7ev9 also contains another copper site belonging to the copper sponge (Lu *et al.*, 2019 ▸). It was not observed in any of Rosenzweig’s cryo-EM structures. The deposited structure contains two copper ions: Cu505 and Cu506 (Fig. 9 ▸*a*). Cu505 has a single ligand, Asp395, which binds using both OD atoms with Cu–O distances of 2.01 and 1.98 Å. Cu506 has only two distant ligands, OG1 of Thr281 and OD1 of Asn306, both at Cu–O distances of 2.70 Å. The Cu–Cu distance is 3.37 Å. The *CMM* validation server suggests that six of the quality measures are dubious and four are borderline, confirming the chemical view that this site is questionable.

We started by performing QR with two copper ions in three different oxidation states [Cu(I)_2_, Cu(II)_2_ and Cu(I)Cu(II)]. The results are shown in Table 7 ▸ and Fig. 9 ▸(*b*). The three structures are quite similar. Cu505 does not move much, with Cu–OD distances of 2.00–2.23 Å. On the other hand, Cu506 moves extensively to form more reasonable Cu–O bond lengths to Thr281A and Asn305A (1.91–2.05 Å). Consequently, the Cu–Cu distance increases to 4.40–5.51 Å. In addition, Cu506 forms a weak interaction with the backbone O atom of Thr281A, with Cu–O distances of 2.59–2.68 Å (3.94 Å in the deposited structure). Naturally, this means that Cu506 moves out of density, which is connected to a large decrease in RSCC for this ion, from 0.73 for the deposited structure to 0.54–0.58 for the QR structures (Supplementary Table S8). The strain energies are also quite large, increasing with the charge of the copper ions (55–263 kJ mol^−1^).

Therefore, we looked for alternative interpretations of the structure. Firstly, we tested replacing one of the copper ions with a water molecule. If we replaced Cu506 with water, more reasonable structures are obtained in which Cu505 coordinates to Asp395 and the water molecule with Cu–O distances of 1.85, 2.83 and 1.91 Å for Cu(I) and 1.99, 2.02 and 1.93 Å for Cu(II). At the same time, the water molecule donates hydrogen bonds to OG1 of Thr281A and OD1 of Asn306A, with H–O distances of 1.73 and 1.98 Å for Cu(I) and 1.76 and 1.90 Å for Cu(II), respectively. The water molecule resides close to the centre of the density, whereas Cu505 moves out of density. This is signalled by a large decrease in RSCC for the copper ion, from 0.56 for the deposited structure to 0.14–0.26 for the QR structures.

If instead Cu505 is replaced by a water molecule, Cu506 binds to OG1 of Thr281A and OD1 of Asn306A, as well as to the water molecule, with Cu–O distances of 1.86–2.47 Å. In both cases, one of the protons of the water molecule moves to Asp395A, forming a copper-bound OH^−^ ion (with a Cu–O distance of 1.86 Å). The protonated Asp395A forms a short hydrogen bond to the OH^−^ ion with a H–O distance of 1.51–1.73 Å. The average RSCC scores are slightly worse than for the other structures: 0.57 for both oxidation states. The strain energies are 43 and 56 kJ mol^−1^, respectively. For Cu(II), a structure without this proton transfer can also be obtained, but it has a higher strain energy (90 kJ mol^−1^) and the same average RSCC of 0.57.

Finally, we tried to replace both copper ions with water molecules. The resulting structure is shown in Fig. 9 ▸(*c*). It can be seen that one water molecule forms two hydrogen bonds to Asp395A (1.64 and 1.93 Å), whereas the other water molecule donates hydrogen bonds to the first water molecule and to Asn306 (1.58 and 2.09 Å) and receives a hydrogen bond from Thr281A (1.60 Å). This structure has the best average RSCC score of all our QR calculations (0.69). A visual inspection also shows good agreement with the map, and both water molecules fit the map. However, the strain energy is relatively large, 107 kJ mol^−1^, but this is only because the unconstrained structure converges to a different hydrogen-bond pattern involving the Asn306 side-chain NH atom. Consequently, our results indicate that this site is better modelled with two water molecules.

## 4. Conclusions

We have studied three recent single-particle cryo-EM structures of pMMO (PDB entries 7s4h, 7s4j and 7ev9; Chang *et al.*, 2021 ▸; Koo *et al.*, 2022 ▸) by means of the recently developed QR approach for cryo-EM (Lundgren *et al.*, 2024 ▸). We assess structures before and after QR by using the RSCC, strain energies and the quality measures of the *CMM* webserver, as well as visual inspection of the resulting structures and ESP maps.

We find that the Cu_A_ (bis-His) site is properly modelled in all three structures. The results indicate that the site is best modelled by a reduced Cu(I) ion. The other copper sites (Cu_B_, Cu_C_ and Cu_D_) in the two Rosenzweig structures also seem to be correctly modelled. However, for Cu_B_ and Cu_D_ there are nearby water molecules with unclear binding properties. One of them (HOH406 of Cu_D_ in PDB entry 7s4h) is stabilized in the second coordination sphere by a hydrogen-bond network. On the other hand, the other water molecules do not form any strong interactions but appear at nonbonding distances in the deposited structures. In the QR structures, they typically move to a binding distance, especially for Cu(II), reflecting that QR will always try to improve all interactions within the QM system if the experimental data allow it. This may indicate that the water molecules show extensive dynamics (as can be expected for a weak axial ligand) or that there is a mixture of oxidized and reduced ions in the sites (the oxidation states of the copper ions in the structures are not known).

On the other hand, our results indicate that the other metal sites modelled in PDB entry 7ev9 are highly questionable. By testing different structural interpretations, we suggest that the Cu_B_ site is better modelled as a mononuclear site, as in the other two cryo-EM structures and in the newer crystal structures (Cao *et al.*, 2018 ▸; Ross *et al.*, 2019 ▸; Ro *et al.*, 2019 ▸). For the other three putative copper sites (Cu_E_, Cu504 and Cu505/506), our results indicate that there is no experimental support for the metal ions. Instead, all three sites are better modelled with one (Cu504) or two (Cu_E_ and Cu505/506) water molecules. A visual inspection already shows that the three putative metal sites are highly questionable, with too few or no ligands at reasonable bonding distances and often with nonbonding atoms or positively charged groups nearby. This is confirmed by the *CMM* web server, which flags all three sites as highly dubious. QR with the metals can provide more reasonable Cu–ligand distances and coordinating geometries, but the sites are still missing a reasonable number of coordinating residues. However, with water, much more convincing hydrogen-bond networks are obtained.

Metals pose a well known problem for the refinement of both crystal and cryo-EM structures (Babai *et al.*, 2024 ▸). Each metal has distinct preferences for bond lengths and geometries that depend strongly on the oxidation and spin state, as well as on the nature of all of the ligands. Therefore, it is very difficult to set up reliable and general restraints for metal sites (Hu & Ryde, 2011 ▸). However, with QM calculations, such restraints are automatically obtained. Therefore, we suggest that QR is the proper way to treat metal sites in crystal and cryo-EM structures (Lundgren *et al.*, 2024 ▸). We think that the present study illustrates this point further.

We observe for all sites that the RSCC score deteriorates with QR. However, as has been discussed previously (Lundgren *et al.*, 2024 ▸), this is expected. Empirical restraints will always deteriorate the fit to the ESP map compared with an unrestrained structure, especially as the map does not change with the model as in X-ray crystal structures. For the other parts of the cryo-EM structure, this is already the case: the structure is a compromise between the experimental term and the restraint, according to equation (1). However, for the metal sites essentially no restraints are used (a van der Waals repulsion term that is effective only at very short distances). When we employ QM restraints in the QR approach, the metal sites will therefore move slightly away from the ESP map, in the same way as all atoms move slightly away from the map when the normal empirical restraints are turned on. In our previous study, we showed that the metal ions do not move more out of density than other atoms in the cryo-EM structure (Lundgren *et al.*, 2024 ▸). Thus, it can be argued that the metal sites were ‘overfitted’ towards the map in the deposited structures and that it is expected that RSCC scores should deteriorate when the QM restraints make the metal sites more chemically reasonable.

Finally, the present study shows how QR can be used to discriminate between different interpretations of various sites of cryo-EM structures. This is one of the most useful and typical applications of QR (Bergmann *et al.*, 2022 ▸).

## Supplementary Material

Selection of wx factor, RSCC values for all residues in all copper sites and CheckMyMetal results for the deposited structures. DOI: 10.1107/S2059798325008356/dez5001sup1.pdf

Coordinate files of all quantum-refined and QM-optimised structures (only QM region; all other atoms are kept as inthe deposited structures. DOI: 10.1107/S2059798325008356/dez5001sup2.zip

## Figures and Tables

**Table 1:** Description of the various structures (deposited or QR) of the Cu_A_ site The table shows the PDB code, the oxidation state of Cu_A_ in the QR calculations, the average RSCC of the four residues in the QM system (the raw data are given in Supplementary Table S2), the strain energy (Δ*E*_str_ in kJ mol^−1^) and bond lengths involving the copper ion (in Å; N1 is ND1 of His48A, N2 is ND1 of His72A and O is OE1 of Gln404A).

PDB code	Cu	RSCC	Δ*E*_str_†	N1	N2	O
7s4h	Deposited	0.90	101/148	2.03	2.04	2.41
	Cu(I)	0.88	15	1.91	1.92	2.34
	Cu(II)	0.87	37	1.95	1.97	2.02
7s4j	Deposited	0.90	84/134	1.95	1.97	2.38
	Cu(I)	0.88	14	1.91	1.93	2.31
	Cu(II)	0.86	42	1.96	1.96	1.98
7ev9	Deposited	0.92	36/38	1.90	1.87	5.21
	Cu(I)	0.91	4	1.89	1.90	
	Cu(II)	0.91	5	1.91	1.91	

† The first number for the deposited structure is for Cu(I) and the second is for Cu(II).

**Table 2:** Description of the various structures (deposited or QR) of the Cu_B_ site The entries in the table are the same as in Table 1 ▸. The individual RSCC scores are given in Supplementary Table S2. N1 and N2 are N and ND1 of His33A, N3 is ND1 of His137A, N4 is NE2 of His139A, and O1 and O2 are O of Wat626 and Wat639. The Cu_B_ site is binuclear in PDB entry 7ev9; therefore, distances for a second Cu ion (Cu2) are given for some of the structures.

				Cu–*X*							Cu2		
PDB code	Cu	RSCC	Δ*E*_str_†	N1	N2	N3	N4	O1	O2	Cu2	N1	N3	N4
7s4h	Deposited	0.81	≥183	2.55	2.06	2.29	2.07	3.46	3.90				
	Cu(I)	0.79	93	3.12	1.98	2.06	1.97	3.08	3.48				
	Cu(II)	0.80	26	2.08	2.00	2.02	2.01	2.54	3.12				
7s4j	Deposited	0.86	>156	2.75	2.11	2.11	2.04	3.46					
	Cu(I)	0.81	85	2.36	1.97	2.13	1.97	3.05					
	Cu(II)	0.81	44	2.14	1.97	2.07	1.99	2.39					
7ev9	Deposited	0.85	>782	1.66	1.59	3.01				2.27	2.63	1.61	1.63
	Cu(I)_2_	0.77	43	2.07	2.01	2.14				2.38		1.95	1.88
	Cu(I)Cu(II)	0.77	43	2.01	1.95	2.10				2.38		1.97	1.85
	Cu(II)_2_	0.77	67	2.06	2.02	2.14				2.41		1.94	1.87
	Cu(I)	0.79	89	1.97	2.21	2.08	2.33						
	Cu(II)	0.77	27	1.98	1.97	2.02	2.14						

† The strain energies for the deposited structures are 183 and 204 kJ mol^−1^ for Cu(I) and Cu(II) in PDB entry 7s4h, 159 and 156 kJ mol^−1^ for Cu(I) and Cu(II) in PDB entry 7s4h, and 782, 825 and 927 kJ mol^−1^ for Cu(I)_2_, Cu(I)Cu(II) and Cu(II)_2_ in PDB entry 7ev9, respectively.

**Table 3:** Description of the various structures (deposited or QR) of the Cu_C_ site in PDB entry 7s4j The entries in the table are the same as in Table 1 ▸. The individual RSCC scores are given in Supplementary Table S4. O1 and O2 are OD1 and OD2 of Asp156C, N1 is NE2 of His160C and N2 is NE2 of His173C.

Cu	RSCC	Δ*E*_str_†	O1	O2	N1	N2
Deposited	0.83	174/218	2.51	2.36	1.99	2.19
Cu(I)	0.80	7	2.32	2.15	1.95	1.93
Cu(II)	0.78	13	2.05	2.00	1.97	1.99

† The first number for the deposited structure is for Cu(I) and the second is for Cu(II).

**Table 4:** Description of the various structures (deposited or QR) of the Cu_D_ site in PDB entry 7s4h The entries in the table are the same as in Table 1 ▸. The individual RSCC scores are given in Supplementary Table S5. O1 is OD1 of Asn227C, N1 is NE2 of His231C, N2 is NE2 of His245C, and O2 and O3 are O of Wat406 and Wat415.

Cu	RSCC	Δ*E*_str_†	O1	N1	N2	O2	O3
Deposited	0.84	495/501	2.21	2.00	1.50	3.81	3.30
Cu(I)	0.82	91	2.26	1.93	2.05	3.28	2.06
	0.83	149	2.14	1.95	1.96	3.35	2.91
Cu(II)	0.83	82	2.09	1.96	2.01	3.32	1.98

† The first number for the deposited structure is for Cu(I) and the second is for Cu(II).

**Table 5:** Description of the various structures (deposited or QR) of the Cu_E_ site in PDB entry 7ev9 The entries in the table are the same as in Table 1 ▸. The individual RSCC scores are given in Supplementary Table S6. N1 is NE2 of His38B, S is SD of Met42B, O3 is OD1 of Asn103B, O1 and O2 are OE1 and OE2 of Glu100B, and H1 and H2 are NE and HH21 of Arg104B. Distances involving water molecules replacing the copper ions are hydrogen bonds.

				Cu301				Cu302		Wat301	
Cu301	Cu302	RSCC	Δ*E*_str_†	N1	S	O3	Cu302	O1	O2	H1	H2
Deposited		0.80	≥1176	3.28	3.37	2.95	4.22		2.58		
Cu(I)	Cu(I)	0.67	403	1.90	2.21		5.59	2.51	1.93		
Cu(I)	Cu(II)	0.68	397	1.95	2.26		5.38	2.13	1.99		
Cu(II)	Cu(II)	0.69	689	1.90	2.29		4.29	1.89	3.32		
		0.67	549	1.90	2.24		5.42	2.30	1.90		
Cu(I)	Wat	0.70	355	1.89	2.23			1.72			
Cu(II)	Wat	0.70	486	1.93	2.24			1.92			
Wat	Cu(I)	0.76	404	2.08		2.45	3.66	1.83	3.46	2.28	2.41
Wat	Cu(II)	0.76	754	2.07		2.30	3.71	1.82	3.74	2.48	2.61
Wat	Wat	0.73	208	2.16		1.89	4.93	1.73	2.73	2.16	

† The strain energies for the deposited structure are 1176, 1246 and 1407 kJ mol^−1^ for Cu(I)_2_, Cu(I)Cu(II) and Cu(II)_2_, respectively.

**Table 6:** Description of the various structures (deposited or QR) of the Cu504 site in PDB entry 7ev9 The entries in the table are the same as in Table 1 ▸. The individual RSCC scores are given in Supplementary Table S7. O1 is OE1 of Glu316A and O2 is OH of Tyr330A. Distances involving water molecules replacing the copper ion are hydrogen bonds.

Cu	RSCC	Δ*E*_str_†	O1	O2
Deposited	0.79	384/398	2.51	3.22
Cu(I)	0.72	248	1.97	3.40
Cu(II)	0.73	305	2.09	3.36
Wat	0.75	108	2.06	2.49

† The first number for the deposited structure is for Cu(I)and the second is for Cu(II).

**Table 7:** Description of the various structures (deposited or QR) of the Cu505/Cu506 site in PDB entry 7ev9 The entries in the table are the same as in Table 1 ▸. The individual RSCC scores are given in Supplementary Table S8. O1 and O2 are OD1 and OD2 of Asp395A, O3 and O4 are O and OG1 of Thr281A, O5 and N1 are OD1 and ND2 of Asn306A and N2 is N of Gly308A. Distances involving water molecules replacing the copper ions are hydrogen bonds.

				Cu505			Cu506				
Cu505	Cu506	RSCC	Δ*E*_str_†	O1	O2	Cu506	O3	O4	O5	N1	N2
Deposited		0.74	≥232	2.01	1.98	3.37	3.94	2.70	2.70	4.27	3.77
Cu(I)	Cu(I)	0.63	55	2.04	2.00	4.47	2.68	1.99	1.91		2.91
Cu(I)	Cu(II)	0.63	164	2.17	2.14	4.40	2.59	2.05	2.00		2.87
Cu(II)	Cu(II)	0.63	263	2.23	2.13	5.51	2.61	2.04	2.00		2.92
Cu(I)	Wat	0.58	56	2.83	1.85	1.91		1.73	1.98		
Cu(II)	Wat	0.63	59	2.02	1.99	1.93		1.76	1.90		
Wat‡	Cu(I)	0.57	43	1.51		1.86		2.47	1.93	2.99	
Wat	Cu(II)	0.57	90	2.13		2.07		2.07	1.92		
Wat‡	Cu(II)	0.57	56	1.73		1.86		2.09	2.00	3.33	
Wat	Wat	0.69	107	1.64	1.93	1.58		1.60	2.09		

† The strain energies for the deposited structure are 232, 389 and 541 kJ mol^−1^ for Cu(I)_2_, Cu(I)Cu(II) and Cu(II)_2_, respectively.

‡ The water molecule is deprotonated to OH^−^ and the proton moves to Asp395A.
