# Supplementary material for: Critical evaluation of three cryo-EM structures of particulate methane monooxygenase by quantum refinement
Source: Acta Crystallogr D Struct Biol. 2025 Oct 8;81(Pt 11):605–20. doi: 10.1107/S2059798325008356 (PMC12576850; doi:10.1107/S2059798325008356)
Supplement: Supplementary file 1 [file d-81-00605-sup1.pdf]

STRUCTURAL
BIOLOGY

Volume 81 (2025)

Supporting information for article:

Critical evaluation of three cryo-EM structures of particulate methane monooxygenase by quantum refinement

Gayathri Yuvaraj, Kristoffer J. M. Lundgren, Elija Veenman, Esko Oksanen and Ulf Ryde

Table S1. Investigation of the optimum value of w_x for the various Cu sites in the three cryo-EM structures. The table shows the strain energy (ΔE_{str} in kJ/mol), the average RSCC (Av), as well as the individual values of RSCC for the residues in the QM region. The selected value of w_x is shown in bold face, whereas the average value selected by Phenix is shown in *Italics*. For the Cu_C site in 7s4j, w_x was selected in our previous study (Lundgren *et al.*, 2024).

w_x	ΔE_{str}	RSCC				
		Av	H48A	H72A	Q404A	Cu
7s4h	Cu_A					
0	14	0.87	0.92	0.87	0.80	0.88
0.1	15	0.87	0.92	0.87	0.80	0.88
1	13	0.87	0.92	0.87	0.80	0.89
3	15	0.87	0.92	0.87	0.80	0.89
4.6	13	0.87	0.92	0.87	0.80	0.89
**10**	15	0.88	0.92	0.88	0.80	0.90
30	24	0.86	0.91	0.85	0.80	0.88
50	38	0.83	0.90	0.80	0.79	0.84
70	47	0.83	0.90	0.80	0.79	0.84
100	68	0.82	0.89	0.79	0.79	0.82
7s4j	Cu_A					
0	12	0.88	0.92	0.88	0.82	0.90
0.1	12	0.88	0.92	0.88	0.82	0.90
1	12	0.88	0.92	0.88	0.82	0.90
3	11	0.88	0.92	0.88	0.82	0.90
6.6	12	0.88	0.92	0.88	0.82	0.90
**10**	14	0.88	0.91	0.88	0.82	0.88
30	33	0.83	0.88	0.86	0.81	0.77
50	50	0.81	0.88	0.85	0.78	0.72
70	62	0.78	0.86	0.84	0.77	0.65
100	87	0.77	0.87	0.83	0.76	0.63
7ev9	Cu_A					
0	4	0.91	0.90	0.87		0.96
0.1	4	0.90	0.90	0.87		0.95
1	4	0.90	0.90	0.87		0.95
1.6	4	0.90	0.90	0.87		0.95
3	4	0.90	0.90	0.87		0.95
**10**	4	0.91	0.90	0.87		0.95
30	32	0.73	0.82	0.69		0.70
50	58	0.72	0.78	0.68		0.68
70	81	0.69	0.76	0.67		0.64
100	352	0.61	0.65	0.67		0.51

7s4h	Cu_B		H33A	H137A	H139A	W626	W639	Cu
0	25	0.81	0.80	0.89	0.92	0.79	0.61	0.88
0.1	25	0.82	0.80	0.89	0.91	0.79	0.63	0.88
1	35	0.82	0.80	0.89	0.92	0.79	0.62	0.88
**3**	26	0.81	0.79	0.89	0.92	0.79	0.61	0.88
5.0	33	0.81	0.78	0.88	0.91	0.79	0.62	0.87
10	34	0.80	0.75	0.86	0.90	0.78	0.64	0.84
30	38	0.76	0.70	0.83	0.89	0.75	0.60	0.80
50	52	0.69	0.62	0.78	0.86	0.65	0.59	0.66
70	72	0.67	0.62	0.75	0.85	0.62	0.58	0.62
100	108	0.66	0.60	0.73	0.84	0.63	0.55	0.58
7s4j	Cu_B							
0	23	0.82	0.74	0.75	0.86	0.90		0.84
0.1	23	0.82	0.74	0.75	0.86	0.90		0.84
1	23	0.82	0.74	0.75	0.86	0.90		0.84
3	23	0.82	0.74	0.75	0.86	0.90		0.84
5.1	23	0.82	0.74	0.75	0.86	0.90		0.84
**10**	23	0.82	0.74	0.75	0.86	0.90		0.84
30	73	0.78	0.68	0.73	0.84	0.87		0.76
50	83	0.73	0.64	0.72	0.81	0.82		0.65
70	106	0.72	0.63	0.72	0.80	0.81		0.63
100	138	0.71	0.63	0.73	0.79	0.80		0.60
7ev9	Cu_B							
0	156	0.71	0.59	0.80	0.82	0.70		0.63
0.1	156	0.71	0.59	0.80	0.82	0.70		0.63
1	157	0.71	0.59	0.80	0.82	0.70		0.63
**3**	156	0.71	0.59	0.80	0.83	0.70		0.63
10	167	0.70	0.58	0.80	0.82	0.68		0.62
30	172	0.69	0.57	0.78	0.82	0.65		0.61
50	192	0.65	0.56	0.75	0.81	0.59		0.52
70	216	0.49	0.46	0.66	0.67	0.42		0.24
100	237	0.59	0.54	0.67	0.78	0.51		0.43
7s4j	Cu_C		A156C	H160C	H173C	Cu		
0	5	0.79	0.82	0.76	0.82	0.77		
0.1	6	0.79	0.82	0.75	0.82	0.77		
1	6	0.79	0.82	0.75	0.82	0.77		
3	6	0.79	0.82	0.75	0.82	0.77		
5.9	6	0.79	0.82	0.75	0.82	0.77		
**10**	7	0.79	0.82	0.74	0.82	0.77		
30	16	0.78	0.82	0.73	0.82	0.75		
50	28	0.78	0.82	0.73	0.82	0.75		
70	38	0.77	0.82	0.73	0.82	0.74		
100	52	0.77	0.81	0.72	0.81	0.74		

7ev9	Cu_E		H38B	M42B	F50B	E100B	N103B	W301	W302
0	230	0.74	0.81	0.71	0.79	0.80	0.81	0.66	0.62
0.1	228	0.74	0.81	0.71	0.79	0.80	0.81	0.66	0.62
1	228	0.74	0.80	0.71	0.79	0.80	0.81	0.66	0.62
**3**	229	0.74	0.81	0.71	0.79	0.80	0.81	0.66	0.62
5.1	219	0.74	0.81	0.71	0.79	0.80	0.81	0.66	0.62
10	254	0.74	0.81	0.71	0.79	0.80	0.81	0.64	0.62
30	261	0.74	0.81	0.71	0.79	0.80	0.81	0.66	0.61
50	282	0.66	0.79	0.68	0.76	0.75	0.79	0.60	0.26
70	298	0.65	0.78	0.67	0.74	0.73	0.77	0.55	0.32
100	354	0.63	0.78	0.68	0.74	0.73	0.77	0.51	0.19
7ev9	$Cu_{505/506}$		T281A	N306A	D395A	W505	W506		
0	108	0.69	0.78	0.88	0.71	0.50	0.60		
0.1	110	0.69	0.78	0.88	0.71	0.50	0.60		
1	111	0.70	0.78	0.88	0.71	0.51	0.60		
**3**	110	0.70	0.78	0.88	0.71	0.51	0.61		
5.1	106	0.69	0.78	0.88	0.71	0.50	0.60		
10	109	0.69	0.78	0.88	0.71	0.50	0.59		
30	108	0.69	0.78	0.88	0.71	0.50	0.59		
50	107	0.68	0.77	0.82	0.72	0.52	0.56		
70	140	0.68	0.77	0.84	0.73	0.51	0.56		
100	209	0.68	0.77	0.82	0.73	0.48	0.61		

Table S2. RSCC scores of the residues in the QM system of the Cu_A site in the various structures (deposited or QR with Cu in different oxidation states).

PDB	Cu	His-48A	His-72A	Gln-404A	Cu
7s4h	deposited	0.94	0.91	0.83	0.91
	Cu(I)	0.93	0.90	0.81	0.88
	Cu(II)	0.92	0.90	0.80	0.84
7s4j	deposited	0.94	0.91	0.85	0.92
	Cu(I)	0.92	0.90	0.82	0.86
	Cu(II)	0.90	0.90	0.82	0.82
7ev9	deposited	0.92	0.90	0.79	0.95
	Cu(I)2	0.91	0.88		0.93
	Cu(II)2	0.91	0.88		0.93

Table S3. RSCC scores of the residues in the QM system of the Cu_B site in the various structures (deposited or QR with Cu in different oxidation states).

PDB	Cu	His-33A	His-137A	His-139A	HOH-626	HOH-639	Cu
7s4h	deposited	0.80	0.93	0.94	0.69	0.58	0.91
	Cu(I)	0.79	0.90	0.92	0.74	0.55	0.85
	Cu(II)	0.77	0.90	0.92	0.76	0.58	0.87
7s4j	dep	0.84	0.92	0.94	0.72		0.93
	Cu(I)	0.73	0.87	0.91	0.82		0.74
	Cu(II)	0.73	0.87	0.91	0.83		0.69
7ev9					Cu_{502}	Cu_{503}	
	deposited	0.79	0.83	0.85	0.90	0.86	
	Cu(I)2	0.55	0.79	0.81	0.82	0.87	
	Cu(I)Cu(II)	0.55	0.79	0.82	0.82	0.86	
	Cu(II)2	0.59	0.79	0.80	0.83	0.83	
	Cu(I)	0.65	0.81	0.84		0.86	
	Cu(II)	0.64	0.80	0.82		0.83	

Table S4. RSCC scores of the residues in the QM system of the Cu_C site in the 7s4j structure (deposited or QR with Cu in different oxidation states).

Cu	Asp-156C	His-160C	His-173C	Cu
deposited	0.83	0.80	0.88	0.80
Cu(I)	0.86	0.77	0.85	0.72
Cu(II)	0.85	0.77	0.84	0.67

Table S5. RSCC scores of the residues in the QM system of the Cu_D site in the 7s4j structure (deposited or QR with Cu in different oxidation states). All residues belong to the C subunit.

Cu	Asp-156	Arg-165	His-173	Phe-177	Asn-227	His-231	His-245	Cu	HOH-406	HOH-415
dep	0.85	0.83	0.86	0.89	0.90	0.89	0.92	0.86	0.71	0.67
Cu(I)	0.87	0.77	0.85	0.88	0.89	0.86	0.90	0.82	0.73	0.64
	0.87	0.78	0.85	0.88	0.89	0.86	0.90	0.84	0.73	0.74
Cu(II)	0.87	0.77	0.85	0.88	0.89	0.86	0.90	0.82	0.73	0.64
	0.87	0.78	0.85	0.88	0.89	0.86	0.90	0.85	0.73	0.64

Table S6. RSCC scores of the residues in the QM system of the Cu_E site in the 7ev9 structure (deposited or QR with Cu in different oxidation states). All residues belong to the B subunit.

Cu_{301}	Cu_{302}	His-38	Ala-41	Met-42	Met-45	Phe-50	Glu-100	Asn-103	Arg-104	Cu_{301}	Cu_{302}
deposited		0.84	0.87	0.75	0.75	0.84	0.81	0.85	0.88	0.70	0.67
Cu(I)	Cu(I)	0.73	0.81	0.64	0.69	0.79	0.74	0.75	0.83	0.42	0.33
Cu(I)	Cu(II)	0.73	0.81	0.65	0.69	0.79	0.74	0.75	0.83	0.40	0.38
Cu(II)	Cu(II)	0.73	0.81	0.63	0.69	0.79	0.75	0.76	0.84	0.37	0.58
		0.73	0.81	0.64	0.69	0.79	0.74	0.75	0.83	0.41	0.29
Cu(I)	Wat	0.74	0.81	0.65	0.68	0.79	0.74	0.75	0.82	0.44	0.56
Cu(II)	Wat	0.74	0.82	0.65	0.69	0.79	0.75	0.76	0.83	0.41	0.60
Wat	Cu(I)	0.79	0.85	0.69	0.73	0.80	0.78	0.80	0.86	0.65	0.66
Wat	Cu(II)	0.79	0.85	0.69	0.73	0.80	0.78	0.80	0.87	0.65	0.69
Wat	Wat	0.77	0.81	0.69	0.69	0.80	0.74	0.77	0.83	0.65	0.53

Table S7. RSCC scores of the residues in the QM system of the Cu_{504} site in the 7ev9 structure (deposited or QR with Cu in different oxidation states).

Cu_{504}	Glu-316A	Tyr-330A	Arg-323A	Cu	Phe-392A	Phe-394A	Arg-400A
deposited	0.88	0.87	0.81	0.60			
Cu(I)	0.88	0.83	0.71	0.44			
Cu(II)	0.88	0.83	0.72	0.48			
Wat	0.86	0.74	0.82	0.55	0.90	0.81	0.74
	0.89	0.72	0.83	0.53			

Table S8. RSCC scores of the residues in the QM system of the Cu_{505}/Cu_{506} site in the 7ev9 structure (deposited or QR with Cu in different oxidation states).

Cu_{505}	Cu_{506}	Thr-281	Asn-306A	Asp-395A	Cu_{505}	Cu_{506}
deposited		0.84	0.88	0.70	0.56	0.73
Cu(I)	Cu(I)	0.68	0.82	0.66	0.39	0.58
Cu(I)	Cu(II)	0.68	0.83	0.67	0.40	0.58
Cu(II)	Cu(II)	0.67	0.81	0.66	0.47	0.54
Cu(I)	Wat	0.71	0.83	0.63	0.14	0.60
Cu(II)	Wat	0.73	0.87	0.64	0.26	0.65
Wat	Cu(I)	0.68	0.79	0.64	0.17	0.56
Wat	Cu(II)	0.67	0.79	0.65	0.15	0.59
		0.68	0.79	0.63	0.19	0.57
Wat	Wat	0.74	0.86	0.67	0.50	0.66

Table S9. Results of the CheckMyMetal validation server for 7s4h. Results that are considered acceptable, borderline, or outlier are marked in green, yellow and red, respectively.

Nr	ID	Res.	Metal	Occupancy	Bfactor(env.)[1]	Atomic contacts	Valence[2]	nVECSUM[3]	Geometry[1,4]	gRMSD(°)[1]	Vacancy[1]
1	A:501	CU	Cu	1.0	55.7 (43.7)	N_4	1.1	0.2	Square Planar	14.9°	0
2	A:502	CU	Cu	1.0	57.7 (40.0)	O_1N_2	1.0	0.21	Square Planar	9.4°	25%
3	C:301	CU	Cu	1.0	91.1 (38.7)	O_1N_2	2.5	0.63	Tetrahedral	8.3°	25%

Table S10. Results of the CheckMyMetal validation server for 7s4j. Results that are considered acceptable, borderline, or outlier are marked in green, yellow and red, respectively.

Nr	ID	Res.	Metal	Occupancy	Bfactor(env.)[1]	Atomic contacts	Valence[2]	nVECSUM[3]	Geometry[1,4]	gRMSD(°)[1]	Vacancy[1]
1	A:501	CU	Cu	1.0	59.6 (47.0)	N_3	1.1	0.25	Square Planar	9.6°	25%
2	A:502	CU	Cu	1.0	56.7 (44.2)	O_1N_2	1.2	0.17	Square Planar	6.3°	25%
3	C:301	CU	Cu	1.0	30.0 (47.8)	O_2N_2	1.0	0.27	Linear	8.7°	0

Table S11. Results of the CheckMyMetal validation server for 7ev9. Results that are considered acceptable, borderline, or outlier are marked in green, yellow and red, respectively.

Nr	ID	Res.	Metal	Occupancy	Bfactor(env.)[1]	Atomic contacts	Valence[2]	nVECSUM[3]	Geometry[1,4]	gRMSD(°)[1]	Vacancy[1]
1	A:501	CU1	Cu	1.0	57.5 (53.7)	N_2	1.3	0.17	Linear	19.5°	0
2	A:502	CU1	Cu	1.0	75.6 (60.4)	N_3	2.6	0.32	Trigonal Planar	15.8°	0
3	A:503	CU1	Cu	1.0	74.5 (54.0)	N_2	2.6	0.38	Trigonal Planar	15.5°	33%
4	A:504	CU1	Cu	1.0	74.7 (53.7)	O_1	0.1	1.0	Poorly Coordinated	N/A	N/A
5	A:505	CU1	Cu	1.0	93.8 (67.3)	O_2	0.8	0.84	Square Planar	23.5°	50%
6	A:506	CU1	Cu	1.0	89.6 (59.6)	O_2	0.12	0.48	Trigonal Planar	2.3°	33%
7	B:301	CU1	Cu	1.0	88.4 (69.8)	O_1S_1	0.06	0.42	Trigonal Planar	11.2°	33%
8	B:302	CU1	Cu	1.0	96.4 (62.9)	O_1	0.08	1.0	Poorly Coordinated	N/A	N/A
